# Engineering Processes for Plant‐Based Meat Analogs: Current Status and Future Outlook

**DOI:** 10.1111/1541-4337.70322

**Published:** 2025-10-31

**Authors:** Saqib Gulzar, Abdul Fateh Hosseini, Olga Martín‐Belloso, Robert Soliva‐Fortuny, Syed S. H. Rizvi

**Affiliations:** ^1^ Department of Food Technology, Engineering and Science University of Lleida Lleida Spain; ^2^ Agrotecnio ‐ CERCA Center Lleida Spain; ^3^ Department of Food Science Cornell University Ithaca New York USA

**Keywords:** consumer acceptance, extrusion, fibrous texture, plant‐based meat analogs, plant proteins

## Abstract

Plant‐based meat analogs (PBMAs) have emerged as a promising alternative to conventional meat, driven by growing consumer interest in sustainability, ethical considerations, and health‐conscious diets. However, despite initial market enthusiasm, PBMAs struggle with declining consumer acceptance due to their inability to fully replicate the texture, juiciness, and sensory experience of animal‐derived meat. Key limitations include insufficient fibrous structure, reduced tenderness, poor moisture retention during cooking, inadequate lipid distribution mimicking marbling, and unsatisfactory mouthfeel. Addressing these challenges is critical for advancing PBMA development and securing long‐term market success. Unlike most reviews that broadly examine PBMA, this review specifically explores the role of processing technologies and equipment designs in enhancing meat‐like properties. In this review, we have analyzed the commonly used technique for creating PBMA, the high‐moisture extrusion (HME), detailing its mechanisms, equipment configurations, and processing parameters. It also briefly covers some other techniques for creating fibrous structures. Furthermore, the critical interplay between equipment parameters, ingredient properties, and final product characteristics, considering factors such as die design, shear force, moisture content, temperature control, and gas incorporation, is also covered. Moreover, we highlight major technical challenges, including scalability, cost‐effective production, molecular interactions during processing, and consumer‐perceived authenticity. Finally, we propose future directions for refining processing techniques, innovating equipment designs, and overcoming commercialization barriers. By bridging existing knowledge gaps and offering practical insights, this comprehensive analysis aims to support researchers and industry professionals in advancing next‐generation PBMA processing technologies for widespread industrial adoption to make more consumer‐acceptable products.

## 1 Introduction

The consumer demand for plant‐based meat analogs (PBMAs) continues to rise, driven by environmental, ethical, and health considerations. However, achieving widespread consumer acceptance is constrained by persistent challenges in replicating the complex sensory attributes of animal meat, particularly its fibrous, anisotropic protein structure, which underpins mouthfeel, chewability, and elasticity. This structure defines the mechanical properties of meat and is inherently difficult to reproduce using plant proteins due to fundamental differences in protein composition and alignment (Vallikkadan et al. 2023). Additionally, intramuscular fat marbling contributes to juiciness and richness, and replicating its stable distribution and visual authenticity in plant‐based matrices remains a significant hurdle. Despite advances in processing technologies, inconsistencies in reproducing the combined effects of protein structuring and lipid integration often result in suboptimal texture and reduced consumer satisfaction (Giacalone et al. 2022; Kim et al. 2024).

Recent advancements in meat analog production have focused on various innovative technologies aimed at enhancing product quality, scalability, and sustainability. Among these, high‐moisture extrusion (HME) remains one of the most widely adopted methods, effectively combining heat, shear forces, and moisture to denature and align proteins into anisotropic structures resembling muscle fibers (See et al. 2025; Zhang et al. 2023). Although highly suitable for large‐scale production, HME often necessitates careful optimization of parameters such as temperature, screw speed, moisture content, and die geometry in order to achieve desirable textural properties consistently (Ahmad et al. 2024). To address some of the limitations associated with traditional extrusion, alternative technologies have been explored. Shear cell technology has emerged as a promising, energy‐efficient approach, capable of producing fibrous structures at lower moisture content, thus offering environmental sustainability benefits (Dekkers et al. 2018). Additionally, extrusion‐based three‐dimensional (3D) printing provides exceptional precision in designing complex textural profiles and intricate structures, though current scalability remains comparatively limited (Maxy et al. 2024; Qiu et al. 2023). Combining these technological solutions with functional additives—such as hydrocolloids, emulsifiers, and oleogels—has further demonstrated potential for significantly enhancing both the structural and sensory qualities of meat analogs (da Silva et al. 2024; Guo et al. 2023; Sivakanthan et al. 2022; Yousefi and Jafari 2019).

While significant progress has been made, considerable challenges continue to constrain growth in the field. A major challenge is the inherent difference in protein structure and composition between plant and animal sources, which complicates the replication of the fibrous, anisotropic texture of meat. There remains a limited understanding of the molecular interactions between plant proteins, lipids, and additives, which are crucial for creating the fibrous structures that replicate the texture of animal meat. Consumer acceptance of PBMA is primarily determined by sensory attributed such as flavor, aroma, and texture, while ethical or environmental claims influence purchasing decisions only when sensory quality meets expectations. Negative perceptions of ultraprocessing and high prices can undermine acceptance, highlighting the need for clean‐label and cost‐effective formulations (He et al. 2020). Additionally, large‐scale production continues to face challenges in achieving consistent textural outcomes, a key factor in consumer acceptance (Liu et al. 2022). Additionally, most studies have focused on traditional proteins like soy and wheat, while novel sources such as chickpeas, lentils, and algae remain underexplored (Kurek et al. 2022). There is also a limited understanding of how specific interactions between proteins, lipids, and additives influence the mechanical and sensory properties of PBMA. Continued challenges also persist in optimizing flavor and juiciness, as off‐flavors and dryness often hinder consumer acceptance. Addressing these gaps requires integrating insights from protein chemistry, process engineering, and sensory science to develop innovative solutions.

This review paper provides a comprehensive analysis of the mechanisms, challenges, and advancements in the formation of fibrous structures in plant‐based protein products. It focuses on novel protein sources, emerging technologies, and the role of functional additives to improve texture, flavor, and other sensory qualities. Additionally, it integrates insights into evolving consumer preferences, emphasizing the importance of aligning product development with expectations for meat‐like textures, juiciness, and flavor profiles, which are critical for widespread adoption. By addressing existing gaps and highlighting recent innovations, this review contributes to a deeper understanding of how plant‐based proteins can be engineered to replicate the complexities of animal meat. It provides a roadmap for future research, ultimately driving the development of sustainable, high‐quality meat analogs that cater to consumer demands while addressing environmental and ethical challenges.

This review was conducted through a comprehensive and structured search of scientific literature published primarily between 2010 and 2025 (March), with emphasis on articles from the last 5 years to ensure recent developments were captured. The following databases were used: Scopus, Web of Science, PubMed, and Google Scholar. Search terms included combinations of keywords such as: “plant‐based meat analogs,” “high‐moisture extrusion,” “fibrous structure,” “plant proteins,” “sensory properties,” “texture engineering,” “extrusion die design,” “3D printing,” “shear cell,” “ingredient functionality,” and “consumer acceptance.” Articles were included based on their relevance to plant‐based meat processing, ingredient structuring, equipment design, and sensory or nutritional evaluation. Both original research articles and review papers were considered, while commercial reports were used selectively to reflect market trends and consumer perspectives. Although priority was given to recent publications, selected older references were also included when they provided foundational insights or essential background relevant to the understanding of key concepts. Priority was given to peer‐reviewed publications indexed in major scientific databases.

## 2 Current Status of the Plant‐Based Meat Industry

The plant‐based meat market, which experienced rapid growth from 2019 to 2021, has recently faced a slowdown, with declining sales in key regions like the United States, where retail sales dropped by 1% in 2022 and unit sales fell by 8%, according to data from the Good Food Institute and SPINS (GFI 2024). This decline is attributed to high prices, taste and texture concerns, perceptions of being overly processed, and competition from whole‐food plant proteins (Michel et al. 2021). Several key players dominate the market, including Beyond Meat, Impossible Foods, Nestlé, Tyson Foods, and Unilever, all of which continue to innovate in formulation, processing technologies, and ingredient functionality (Szczepkowska 2024). Startups and research institutions are also contributing significantly to the development of novel protein sources, including pea, fava bean, chickpea, and mycoprotein‐based meat analogs (Vila‐Clarà et al. 2024). Between 2021 and 2023, US sales of plant‐based meat and seafood products experienced a significant decline. The unit sales dropped by 28% during this period, reflecting a decrease from 270 million units in 2022 to 195 million units in 2024 (GFI 2024). The dollar sales fell 12% and unit sales dropped 19%, resulting in a market share of 1.8% for packaged meat sales (or 0.9% of the total meat category, including nonpackaged meat). While markets in Asia‐Pacific, particularly China and India, show growth potential due to rising incomes and environmental awareness, regions like North America and Europe are experiencing saturation and waning hype (Euromonitor International 2023). Moreover, they reported that the common and the most significant reason shared by those who avoid both plant‐based meat and dairy is a lack of enjoyment in the taste, with this factor outweighing all other motivations for avoiding plant‐based meat by nearly a 3:1 ratio. Studies have shown that consumer ratings of PBMA are significantly influenced by their ability to mimic the texture of traditional meat products (Sha and Xiong 2020). Consumer studies reveal substantial variability in acceptance rates across demographics and regions, emphasizing the need for targeted product development. Andreani et al. (2023) reported that younger generations exhibit higher acceptance of PBMA, driven by environmental consciousness and curiosity about new food experiences. Nevertheless, traditional meat consumers often express reluctance due to perceived gaps in flavor, texture, and overall sensory experience compared to their animal‐derived counterparts (Fiorentini et al. 2020). Hwang et al. (2020) found that 74% of consumers dissatisfied with PBMA cited textural differences as their primary concern. Younis et al. (2023) demonstrated a 22% increase in consumer satisfaction when hydrocolloids were incorporated to improve juiciness and texture. Fibrous texture arising from the anisotropic alignment in meat analogs significantly improved sensory ratings in controlled trials (Chiang et al. 2019). The fibrous texture of PBMA plays a pivotal role in its market success, as the texture is one of the most critical sensory attributes influencing consumer perception and repeat purchases (Rosso et al. 2024). Consumers often benchmark plant‐based meats against the sensory qualities of high‐quality conventional meat, such as desirable bite, chewiness, and mouthfeel, and products that fail to approximate these characteristics are frequently perceived as inferior alternatives. Several studies reported in the literature indicate that a well‐structured fibrous network enhances juiciness and protein alignment, leading to higher acceptance rates among flexitarian and meat‐eating consumers (Dagevos 2021; Michel et al. 2021; Rosso et al. 2024; Xiong 2023). He et al. (2020) noted that many studies report textural or microstructural data without linking them to sensory preference, creating a gap between lab metrics and consumer liking. They call for future work to integrate sensory, nutritional, and life cycle data to ensure both consumer trust and product success.

The global rise in PBMA has been most pronounced in Western, high‐income countries, where these products are often positioned as solutions to environmental degradation from livestock, health risks linked to red and processed meat, and ethical concerns surrounding animal welfare (Godfray et al. 2018; Poore and Nemecek 2018). However, these priorities are not universal. In low‐ and middle‐income countries (LMICs), the central challenge is not overconsumption of animal products, but persistent food insecurity and undernutrition. According to the most recent FAO estimates, approximately 735 million people suffered from chronic hunger in 2022, with the majority living in Sub‐Saharan Africa and Southern Asia (FAO et al. 2023). By contrast, PBMA products currently available are often more expensive than traditional plant‐based foods or even conventional meat in some low‐income markets (Santo et al. 2020; Bryant 2022). Their production and distribution require advanced infrastructure, such as cold storage, reliable electricity, and efficient supply chains, which are frequently insufficient in resource‐constrained settings (Tziva et al. 2020). Nutritionally, while PBMA can be a good source of protein, it may not provide the full range of micronutrients found in animal products unless they are specifically fortified, and their health benefits can be overstated if processed ingredients and additives are relied upon (Bohrer 2019). Additionally, PBMA products designed for Western consumers may not align with local food preferences or cultural norms in LMICs (Majcher 2025). Thus, their direct relevance to alleviating hunger and malnutrition in the world's most vulnerable regions remains limited. Nonetheless, plant‐based innovation could still play a role in global food security if future efforts shift toward developing affordable, nutrient‐dense plant‐based foods that leverage indigenous and orphan crops and local food traditions. Fortifying plant‐based foods with essential micronutrients would further increase their value in countries where deficiencies are widespread. Achieving these goals will require not only technological innovation but also public–private partnerships, supportive policies, and research that centers on the needs and preferences of food‐insecure populations (Herforth et al. 2020).

## 3 Processes for Engineering Fibrous Texture

Extrusion is the primary process for engineering fibrous texture in PBMAs, as it effectively transforms plant proteins into structured, meat‐like fibers (Dinali et al. 2024). By applying heat and shear forces in the presence of water, extrusion aligns protein molecules into anisotropic structures that replicate the fibrous nature of animal muscle. HME is particularly effective in producing whole‐muscle analogs, while dry extrusion is better suited for granular or ground meat textures (Schmid et al. 2022). The process allows for precise control over texture, density, and moisture retention, making it a cornerstone technology in the development of plant‐based meat alternatives. Additionally, emerging technologies such as 3D printing, shear cell technology, and electrospinning are being explored to further enhance textural properties and expand the possibilities for meat analog production.

### 3.1 Extrusion‐Based Technologies

#### 3.1.1 Wet Extrusion or HME

**FIGURE 1:**
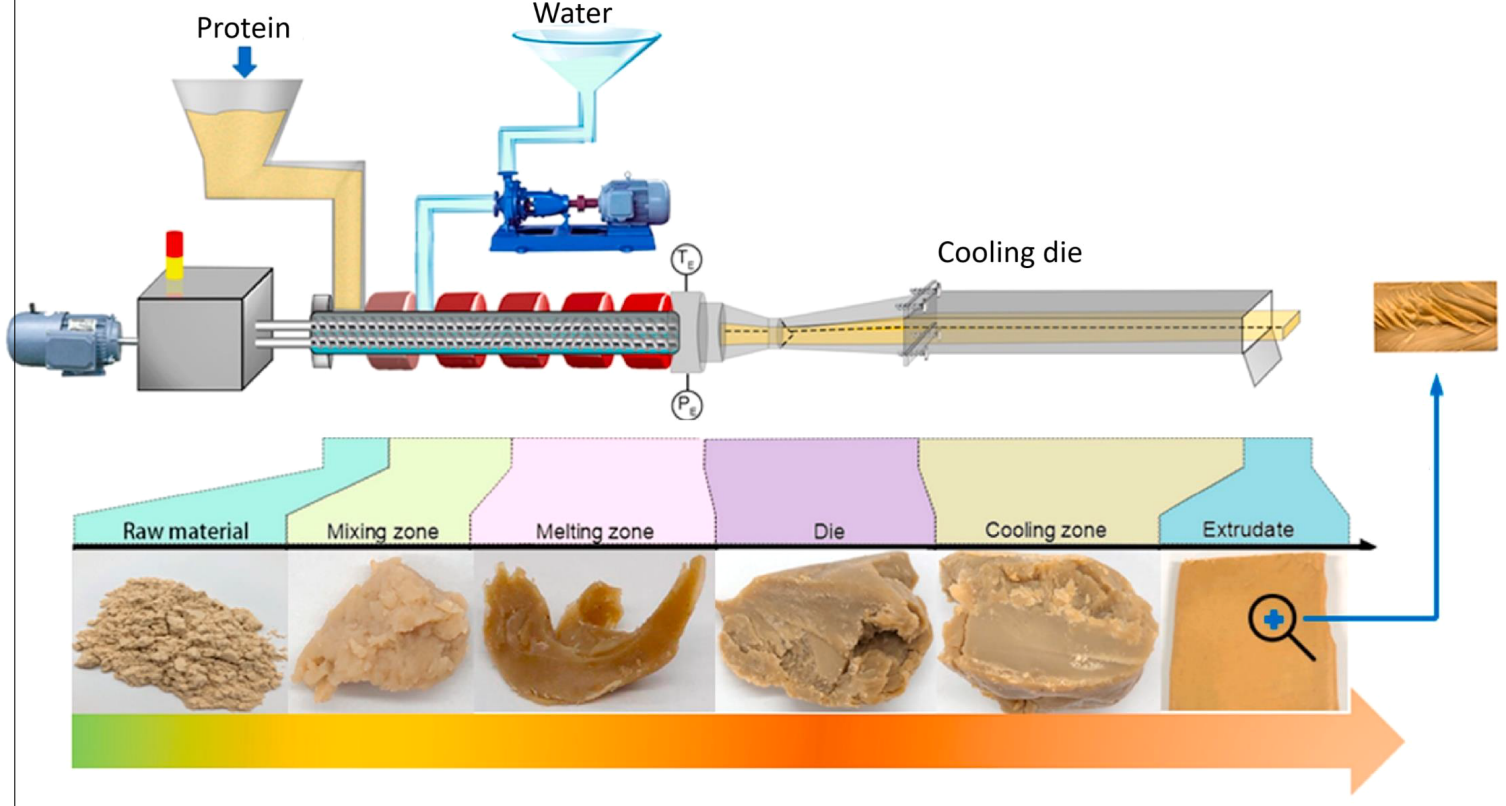
Schematic illustration of the high‐moisture extrusion process. Adapted from Chen et al. (2022).

Wet extrusion is a high‐moisture processing method used to transform plant‐based proteins into fibrous structures that closely replicate the texture and anisotropic properties of animal muscle (Dinali et al. 2024). In this process, a heterophase blend of often incompatible water‐plasticized biopolymers (proteins and some starch) is brought to high temperatures and pressures. The process begins with a thermoplastic transition, wherein proteins denature under the influence of heat, mechanical energy, and moisture. In this step, proteins lose their native globular conformation as heat unfolds their polypeptide chains, exposing internally buried hydrophobic and reactive groups that engage in intermolecular interactions (Guyony et al. 2023). Water, at a high concentration of 40%–80%, acts as a plasticizer, softening the protein matrix and increasing its molecular mobility. This plasticized state allows proteins to flow and deform under applied shear forces, forming a molten matrix suitable for restructuring (Chen et al. 2010). As the molten mass advances through the extruder, shear‐induced alignment occurs, a critical stage in forming fibrous textures. Shear forces generated by the rotation of screws and the barrel's constriction align protein molecules in the flow direction. This molecular alignment is pivotal for achieving anisotropic structures that replicate the striated texture of muscle fibers (Cornet et al. 2022). During this phase, reformation of covalent bonds, such as disulfide bridges, and noncovalent interactions, including hydrogen bonds and hydrophobic associations, reinforces the aligned protein network (Nasrollahzadeh 2023). These interactions stabilize the structure, giving it the mechanical properties necessary to mimic meat textures. Additionally, the viscoelastic behavior of the molten protein matrix facilitates the transition from a disordered to an ordered, fibrous network, which is essential for achieving a realistic meat‐like texture (Sun et al. 2022). The schematic of a typical extruder setup is shown in Figure 1.

The cooling‐induced stabilization phase follows as the extrudate exits the barrel and enters the cooling die. This phase involves rapid temperature reduction, which solidifies the aligned protein structure by locking the molecular interactions formed during the shear stage. The cooling die not only stabilizes the fibrous structure but also shapes the product, ensuring uniformity and texture consistency. The redistribution of moisture that occurs throughout the extrudate during this phase prevents shrinkage and enhances its mechanical integrity. Improper cooling can result in the collapse or deformation of the fibrous structure, making this step critical to the quality of the final product (Nasrollahzadeh 2023; Zhang et al. 2023). Phase separation between protein and water further contributes to fibrous structure formation during wet extrusion, where the protein forms a continuous phase, while water acts as the dispersed phase, facilitating the development of anisotropy under applied shear forces. The extent of phase separation depends on the protein source, extrusion temperature, and shear intensity. Soy proteins exhibit superior phase separation properties compared to other plant proteins, such as peas and lentils, due to their robust water‐binding and gelation characteristics (Zhang et al. 2021). Additionally, innovations in cooling die design have played a pivotal role. Dynamic cooling systems enable precise temperature control during the stabilization phase, locking protein alignments into place and preventing deformation. Such advancements ensure consistency and quality in the final product. Multistage extruders, with independently controlled segments, allow simultaneous execution of hydration, denaturation, and alignment processes, offering unprecedented control over the complex transformations required for the formation of fibrous structures (Zhang et al. 2023).

#### 3.1.2 Dry Extrusion or Texturization

**FIGURE 2:**
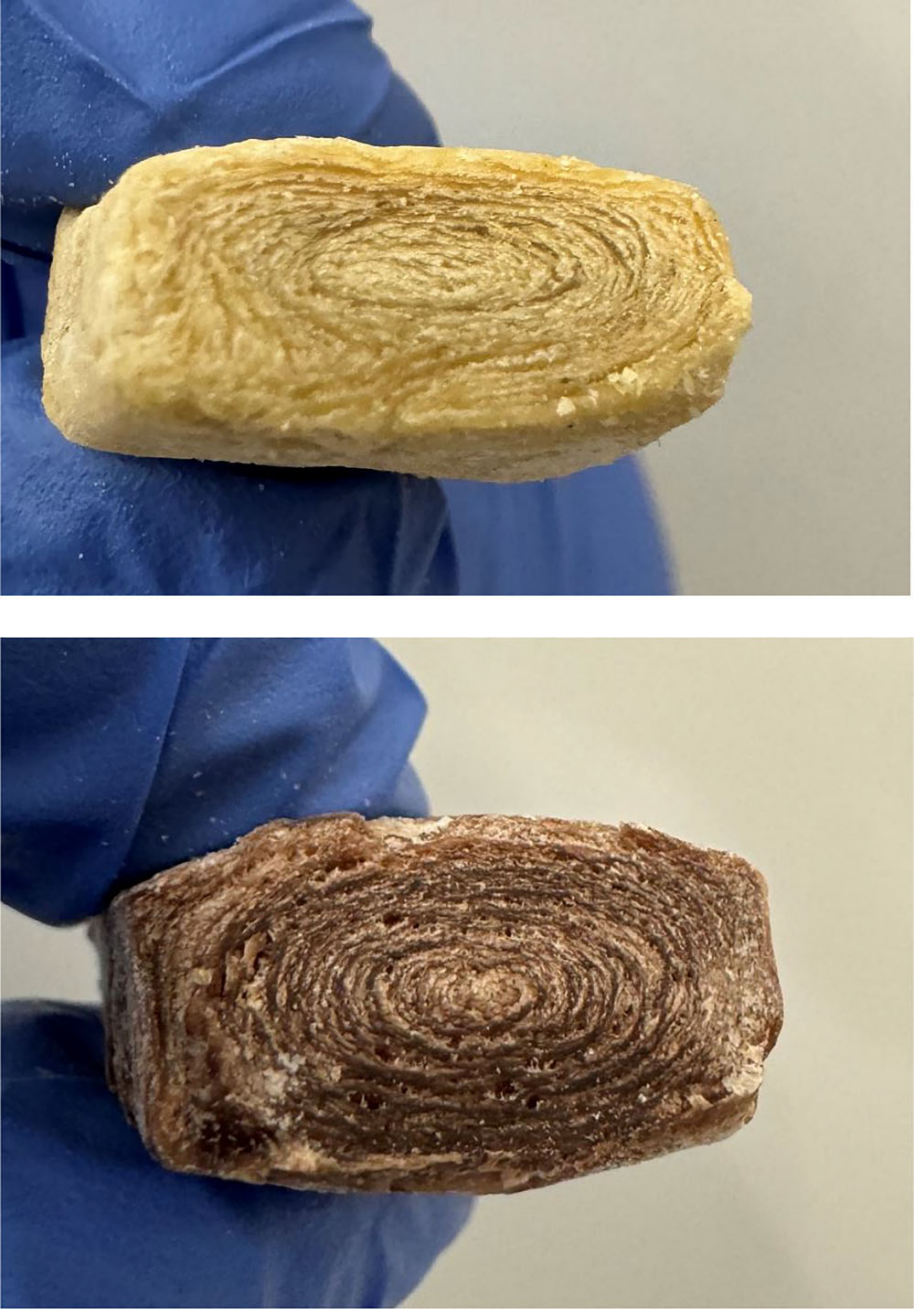
Freeze‐dried soy protein extrudates displaying characteristic layered structures indicative of fibrous texture development.

Dry extrusion, while less prevalent than wet extrusion, plays a significant role in the formation of specific textures and is particularly suited to certain plant‐based products. This method operates under a similar principle to wet extrusion but with a crucial difference: the moisture content in the protein mixture is drastically reduced, usually to less than 15% (Miller 1985). This low‐moisture environment significantly affects the protein alignment process during extrusion. In dry extrusion, the lower water level reduces the plasticizing effects typically provided by moisture, forcing the proteins to align under the influence of high temperature and mechanical shear alone (Vatansever and Hall 2024). This environment creates a distinct texture in the extruded product. Specifically, the resulting texture tends to be chewier and less juicy when compared to products made via wet extrusion, mirroring certain desired qualities for specific food products like snack bars or cereals that benefit from a firmer texture. The low‐moisture content in the protein matrix during the extrusion process increases friction within the extruder barrel and provides the necessary thermal and mechanical energy to unfold protein molecules, forming a disordered but pliable matrix (Arêas 1992). Under shear forces, these unfolded proteins align and aggregate, stabilizing into layered, fibrous structures as the material exits the die, as can be seen in Figure 2. The critical variables in dry extrusion are temperature and mechanical shear. Without adequate moisture to facilitate plasticization and cooling processes, precise control of these parameters becomes critically important for successful dry extrusion. Optimizing temperature and shear levels is key to achieving the desired texture and ensuring that the protein structures align effectively with the limited amount of water available to mediate the process (Orias et al. 2002). This makes dry extrusion a specialized, yet valuable, method for achieving specific textural outcomes in producing plant‐based foods.

Soy and pea proteins (PPs) are the most used matrices for dry extrusion due to their strong thermal stability and protein functionality. Soy protein, with its high content of globular proteins, is particularly suited to creating fibrous textures, as it forms robust networks through hydrophobic interactions and disulfide cross‐links (Zheng et al. 2022). PP, while less elastic, is increasingly used in blends to enhance the sustainability and allergen‐free nature of dry‐extruded products. Recent studies also highlight the potential of incorporating functional additives such as dietary fibers and hydrocolloids during extrusion to improve water retention, elasticity, and overall texture (Marczak and Mendes 2024; Zhang et al. 2024c). The primary advantage of dry extrusion is its energy efficiency and cost‐effectiveness compared to HME (Rokey et al. 2010). The process eliminates the need for extensive drying postextrusion, making it more suitable for large‐scale production of shelf‐stable products such as granules, flakes, and crumbles. These products are particularly versatile for use in soups, sauces, and processed plant‐based meats (Ma et al. 2018). However, the lower moisture content in dry extrusion also imposes challenges. It limits the plasticity of the protein matrix, reducing molecular mobility and resulting in less anisotropic structures than wet extrusion. Consequently, dry‐extruded products are typically better suited for applications requiring coarse textures, such as ground meat analogs, rather than whole cuts (Nisov 2024).

#### 3.1.3 Molecular Changes Occurring During Extrusion

Extrusion cooking induces key molecular transformations in plant‐based proteins, critical for fibrous texture development in meat analogs. These molecular changes are initiated and shaped by the thermal and mechanical conditions within the extruder, and they can be categorized into three interrelated processes: protein denaturation, alignment, and bonding (Verbeek and Van Den Berg 2010). Together, these transformations create a stable, fibrous structure that mimics the muscle fibers of animal meat. Protein denaturation is the first and most critical step in extrusion cooking. Under the influence of high temperatures (typically 100–180°C) and intense shear forces, the native structure of proteins is disrupted. This process unfolds protein molecules from their globular forms into extended chains, exposing their hydrophobic regions to the environment (Schmid et al. 2022). These newly exposed sites play a crucial role in subsequent molecular interactions, as they increase the potential for protein–protein and protein–water interactions. The extent of denaturation depends on factors such as the protein type, extrusion temperature, and residence time in the extruder. Soy proteins, with their high globulin content, require precise control of thermal and mechanical energy to achieve complete denaturation without degradation (Sui et al. 2021). Wheat gluten (WG), on the other hand, denatures more readily due to its inherent elasticity and responsiveness to heat (Abedi and Pourmohammadi 2021).

HME is a complex process characterized by protein aggregation and phase separation, which are pivotal for forming the fibrous texture of high‐moisture meat analogs (HMMAs). Protein denaturation and alignment during extrusion alter the protein's conformation, exposing hydrophobic regions that interact with flavor compounds, thereby influencing flavor retention and perception (Akdogan 1999). In addition to physical transformations, high temperatures in the extrusion barrel facilitate chemical reactions such as the Maillard reaction and lipid oxidation, which contribute to flavor development (Chakraborty et al. 2014). These reactions can generate desirable roasted or caramelized notes but may also produce off‐flavors if not carefully controlled. Moreover, the extrusion process modulates the matrix of raw materials, affecting the release of volatile compounds critical to flavor perception. Variations in processing conditions, such as temperature, shear rate, and feed composition, directly influence these molecular interactions and the sensory characteristics of the final product (Zhang et al. 2019). The role of proteins, starches, fibers, and other ingredients in HME for plant‐based meat, detailing how extreme conditions induce conformational changes and molecular interactions (disulfide, hydrogen, hydrophobic bonding) that form fibrous meat‐like structures. Chicken analogs with anisotropic structures (index > 1) were created using soy protein isolate (SPI), WG, and whey protein (WP) concentrate blends via HME, achieving a texture similar to cooked chicken breast through noncovalent interactions (hydrogen and hydrophobic bonding) and secondary structures (β‐sheets, α‐helix, β‐turns; Tan et al. 2024).

Recent molecular analyses show that fibrous texture in PBMA arises more from flow‐guided phase morphology—where protein‐rich continuous phases and water‐rich domains elongate under shear—and are subsequently stabilized by hydrogen bonding, hydrophobic interactions, and disulfide interchange during cooling (Ahmad et al. 2024). As the denatured proteins move through the extruder, they are subjected to mechanical shear forces, which play a pivotal role in aligning the unfolded protein molecules. These forces, generated by the screws and barrel configuration, orient the protein chains in the direction of the material flow (Day and Swanson 2013). This alignment is critical for replicating the fibrous, linear arrangement of muscle tissue of animal meat. The degree of alignment is influenced by factors such as the shear rate, screw speed, and die geometry. High shear rates and optimized screw speeds enhance molecular alignment by elongating the protein chains along the extrusion axis. Under high moisture and elevated temperature, proteins unfold and expose hydrophobic patches and thiol groups; during cooling these reactive sites promote interchain bonding that reinforces the oriented structure (Ahmad et al. 2024). Additionally, the narrow exit of the extrusion die intensifies this alignment by focusing on the flow direction. The aligned proteins serve as the structural framework for the fibrous texture, providing the anisotropy needed to mimic meat fibers (Ribeiro et al. 2024). However, the formation of a visibly fibrous, anisotropic structure, critical for mimicking muscle fibers, occurs primarily in the cooling die (Guan et al. 2024). Upon exiting the extruder, the aligned proteins encounter cooler temperatures and reduced pressure. These changes in environmental conditions promote the formation of stable chemical bonds between the protein chains, solidifying the fibrous network. Two primary types of bonding occur during this stage:

1. Covalent bonds: Disulfide bridges form between sulfur‐containing amino acids, providing strong, permanent linkages that reinforce the protein matrix (Beniwal et al. 2021).
2. Noncovalent interactions: Hydrogen bonds, ionic bonds, and hydrophobic interactions further stabilize the structure. These bonds contribute to the cohesive and elastic properties of the fibrous texture, enhancing the mechanical integrity of the final product (Chen et al. 2011; Osen et al. 2015).

The nature and strength of these bonds are influenced by the protein composition and the cooling rate. Generally, slower cooling promotes more extensive bond formation, resulting in firmer and more cohesive textures. The inclusion of additives such as transglutaminase can further enhance cross‐linking, improving the structural stability and chewiness of the final product (Yu et al. 2025). Adding WP to pea protein isolate (PPI) enhances the formation of fibrous structures during HME. WP improves textural properties, increases disulfide bonds, and promotes β‐sheet structures, resulting in more compact aggregates and anisotropic textures, making it a promising modifier for PPI‐based meat analogs (Zhang et al. 2024c). Soybean–wheat coprecipitation protein (SWCP) produced superior fibrous textures in HME compared to blend proteins, with extrusion parameters and molecular changes significantly affecting texture formation (MacQueen et al. 2019). Faba bean protein isolate (FPI) and faba bean protein concentrate (FPC) blends can produce fibrous meat analogs using HME, with higher FPC content enhancing fiber formation. Increased FPI content improved textural properties like hardness and chewiness but reduced water absorption and fibrous organization (Kantanen et al. 2022). The addition of β‐glucan (BG) to SPI extrudates under low‐moisture extrusion enhanced disulfide and hydrogen bonding, improving oil‐holding capacity and cooking yield while reducing water solubility, which aids nutrient retention (Xiao et al. 2023). At 5% BG, the extrudates formed large molecular weight compounds stabilized by these interactions. Moreover, the modification of soy protein‐based materials with gelatin, lactose, and sucrose under low‐moisture extrusion revealed significant effects on processability and product properties. Gelatin increased specific mechanical energy (SME) but disrupted product continuity, while lactose reduced SME and promoted Maillard reactions, leading to compact, ordered structures, as confirmed by FTIR, XRD, and SEM analyses (Guerrero et al. 2012). Incorporating insoluble dietary fiber (IDF) into SPI enhances the fibrous structure and mechanical anisotropy of high‐moisture extrudates. At 10%–20% IDF substitution, a mesh‐like SPI/IDF matrix forms, promoting fibrous appearance and structural strength, while excessive IDF causes phase separation and structural collapse (Deng et al. 2023). Dry‐fractionated PP (PDF) enables sustainable, clean‐label meat analogs with 55% protein content, producing porous, softer extrudates with high oil absorption. Though requiring higher extrusion energy, PDF offers unique sensory profiles compared to neutral protein isolates (De Angelis et al. 2020).

#### 3.1.4 Factors Affecting the Extrusion Process

The formation of fibrous textures in PBMAs is highly dependent on the processing conditions during texturization. The extrusion parameters, pH and ionic strength, and moisture content are key variables that influence the structural alignment and stabilization of plant proteins, ultimately dictating the quality and sensory attributes of the final product (Alam et al. 2016; Chaiyakul et al. 2009). In extrusion‐based texturization, several factors, including barrel temperature, screw speed, feed moisture content, and die shape, are critical in determining the alignment and strength of the protein network (Zhang et al. 2023). Higher temperatures during extrusion facilitate protein denaturation, exposing hydrophobic and reactive groups that promote intermolecular interactions. The application of shear forces via screw rotation aligns these denatured proteins into anisotropic structures, mimicking the fibrous nature of animal muscle. Studies have shown that increasing the screw speed enhances shear intensity, leading to improved alignment and a more uniform fibrous structure. However, excessive shear can degrade proteins, compromising texture quality (Ribeiro et al. 2024). Osen et al. (2014) highlighted that HME of PPI (83%–85% protein content) requires barrel temperatures above 120°C to induce full protein denaturation, which is critical for forming fibrous structures in meat analogs. Higher temperatures (up to 160°C) expose hydrophobic groups, promoting protein–protein interactions, and phase separation necessary for fibrous texture development. Further analysis revealed that protein denaturation, marked by a reduction in α‐helices and β‐sheets and an increase in protein aggregates, stabilizes the product's structure, demonstrating parallels between low‐ and HME in protein conformational changes. Texturization techniques, such as stretching, rolling, or folding protein dough, further enhance fibrous texture formation by physically aligning protein structures in parallel arrangements. These methods are commonly employed in downstream processing, after initial protein structuring, to refine the texture and replicate the layered properties of muscle tissue. Stretching protein dough elongates the protein network, encouraging the formation of ordered, anisotropic structures. Folding and laminating techniques, on the other hand, layer the protein matrix repeatedly, enhancing the mechanical strength and chewiness of the final product (Kinsella and Franzen 1978). Such techniques are widely used in traditional texturization methods, such as those applied to WG‐based seitan, to create chewy, fibrous meat alternatives (Singh et al. 2024).

##### 3.1.4.1 Effect of Die Design and Cooling Die

**FIGURE 3:**
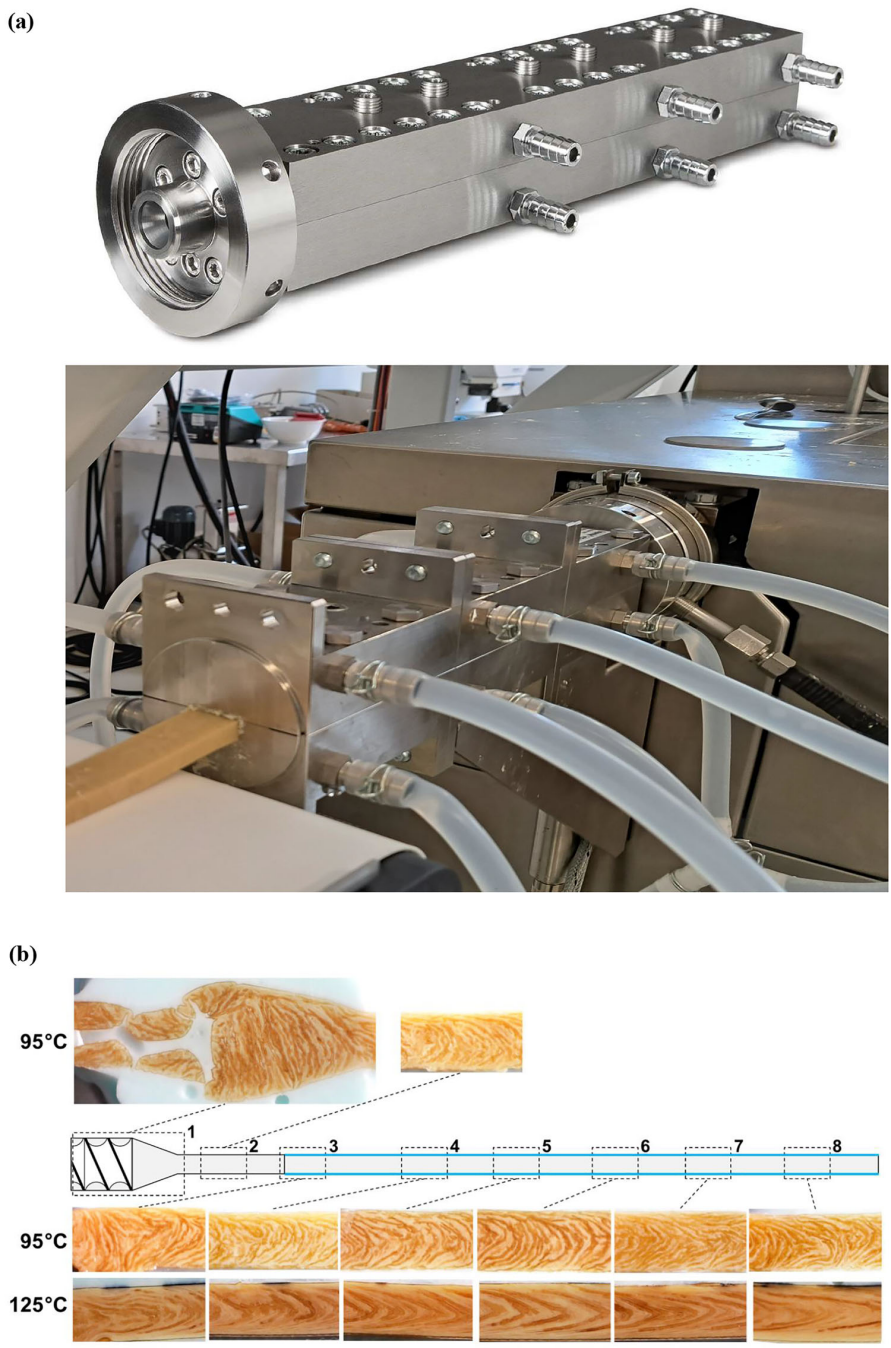
(a) Image of a typical cooling die used in a twin‐screw extruder, TwinLab‐F 20/40 (Brabender GmbH & Co., Duisburg, Germany); (b) Morphology of soy protein samples obtained through cryo‐imaging during dead‐stop trials at material temperatures of 95°C and 125°C, taken from different locations within the die section. Flow direction is from left to right. Images adapted from Wittek et al. (2021b).

During HME, the cooling die plays a pivotal role in establishing the fibrous, anisotropic texture of meat analogs, not merely by lowering temperature, but by shaping the flow dynamics and microstructure as the material solidifies, as shown in Figure 3a. It facilitates gradual solidification and directional protein alignment essential for fibrous structure formation. Unlike the barrel section, where protein denaturation and plasticization occur, the cooling die is primarily responsible for shaping the anisotropic structure through laminar flow and controlled solidification. The transition of the protein matrix from a molten to a solid state within the cooling die determines the preservation of fluid‐mechanical stresses, which are essential for the development of anisotropic, fibrous structures resembling animal meat (Högg et al. 2017; Wittek et al. 2021b). Wittek et al. (2021a) demonstrated using SPI that the fibrous morphology results not from protein chain orientation alone but also from the alignment of multiphase systems under shear and tensile forces generated in the cooling zone. Their study highlighted that tensile stresses of approximately 100 kPa and shear stresses near 400 kPa are produced at critical points within the die, especially in the transition zone, facilitating alignment of protein‐ and water‐rich domains which subsequently “lock in” as the material cools. Sandoval Murillo et al. (2019) further explained that spinodal decomposition leads to the development of phase‐separated structures during cooling, where the elongation of water‐rich domains within the protein matrix results in visible fibrous texture. These aligned domains remain stable upon cooling, forming a layered structure essential for mimicking meat fibers. The local flow fields within the cooling die, specifically the distribution of shear and tensile stresses, are closely linked to the formation of the characteristic multilayered structures observed in high‐quality meat analogs as shown in Figure 3b (Wittek et al. 2021b). The magnitude and distribution of these stresses, modulated by die geometry and process parameters such as cooling rate and die temperature, have direct impacts on texture indices like anisotropy and structuring index, which quantitatively reflect fiber alignment and textural fidelity to animal meat (Pietsch et al. 2019; Högg et al. 2025).

Die geometry also significantly impacts texture development. Narrower or specialized die shapes focus on shear forces and protein alignment at the extrusion exit, enhancing the fibrousness and mechanical strength of the final product. These parameters must be optimized for specific protein formulations to balance texture formation, energy efficiency, and scalability (Verfaillie 2024). The pH and ionic strength of the protein mixture influence protein solubility and the electrostatic interactions that govern network formation (Rizvi 1976). Proteins have minimum solubility at their isoelectric point, where electrostatic repulsion is minimized, promoting aggregation and network formation. Slightly acidic or neutral pH conditions are often optimal for texturization as they allow for balanced protein solubility and interaction dynamics. Soy and wheat proteins exhibit enhanced gelation and alignment properties at pH values around 6–7 (Webb et al. 2023). The ionic strength of the protein solution, determined by the presence of salts or other charged molecules, can also modulate protein behavior. Increasing the ionic strength can enhance hydration forces, which may lead to stronger electrostatic repulsion between protein molecules and potentially offset any gains in hydrophobic interactions (Schuldt et al. 2014). Wittek et al. (2021b) explored various die lengths and taper angles and concluded that smaller die diameters and longer lengths enhance elongational flow, thereby improving fiber orientation and mechanical anisotropy. Narrower die widths and optimized cooling profiles have been shown to enhance fiber orientation and increase the anisotropy index, correlating with improved chewiness and juiciness in sensory evaluations (Guyony et al. 2022; Pietsch et al. 2019). Furthermore, the cooling die's ability to rapidly stabilize the matrix prevents undesirable expansion and phase coalescence, ensuring a dense, layered structure that translates into a meat‐like mouthfeel (Högg et al. 2017, 2025). Die geometry and cooling profile act as critical molecular selectors: the transition zone at the die entrance provides tensile and shear stress fields that align phase components before viscosity rapidly rises and locks in structure (Ahmad et al. 2024). However, excessive cooling or suboptimal flow profiles can lead to insufficient alignment or heterogeneous textures, ultimately compromising consumer acceptability (Sandoval Murillo et al. 2019). Recent advances in modeling and experimental validation have enabled the prediction and control of local flow, temperature, and solidification phenomena, allowing for targeted process design to tailor sensory and mechanical properties (Högg et al. 2025). Their models showed that, as expected, surface regions cool faster and become more viscous, restricting flow, while the core remains more fluid, enabling elongation and alignment along the extrusion axis. The resulting velocity gradient promotes the stratification of phases, reinforcing directional strength. Lee et al. (2024) reported that an optimal die exit temperature of 50°C produced SPI extrudates with cutting strength and fibrousness levels most comparable to chicken meat. When the exit temperature deviated from it, the extrudates either exhibited insufficient fiber formation (at high temperatures) or poor cohesiveness and crumbling texture (at overly low temperatures), reaffirming the necessity of thermal control in the die.

##### 3.1.4.2 Effect of Moisture Content

Moisture content is one of the most critical factors in achieving fibrous textures during processing. Water acts as a plasticizer, lowering the viscosity of the protein matrix and facilitating molecular mobility and alignment under shear forces (Gulzar et al. 2025a). In HME, the presence of sufficient water allows for better plasticization and phase separation, resulting in well‐defined anisotropic structures. However, too much moisture can dilute protein interactions, weakening the fibrous network (Gulzar et al. 2025b). Conversely, low‐moisture conditions limit protein mobility, hindering alignment under shear and producing dense, less fibrous textures (Ribeiro et al. 2024). Balancing moisture levels is crucial to achieving the desired texture. The ideal moisture content varies depending on the protein source, with soy proteins typically requiring higher moisture for optimal plasticization compared to WG or PPs. Moisture also influences the thermal and mechanical properties of the extrudate, affecting postprocessing stability and consumer perception of texture (Verfaillie 2024). Wittek et al. (2021a) demonstrated that anisotropic texture forms only when moisture exceeds 40%, combined with temperatures above 100°C and use of a cooling die. Their microstructural analysis revealed a protein‐rich continuous multiphase rather than molecular protein alignment. A recent study on high‐moisture SPI extrudates (55%–75% moisture) found that the anisotropy index peaked at 65%, aligning with the highest β‐sheet content (Zang et al. 2025). Moisture beyond 65% led to a decline in textural hardness due to overplasticization. Zhao et al. (2023) demonstrated that moisture content significantly influences the structural and sensory‐relevant properties of calcium caseinate‐based meat analogs. Increasing the moisture from 60% to 70% improved the fibrous degree from 1.02 to 1.64, indicating stronger anisotropic texture. At 60%–65% moisture, the extrudates closely matched the cutting force and fibrousness of cooked fish such as cod and salmon, suggesting favorable mechanical and sensory qualities. However, higher moisture content reduced hardness and chewiness, indicating a trade‐off between tenderness and structural integrity. The study also highlighted that appropriate cooling die temperatures (45°C) combined with optimal moisture promote gradual setting and better alignment. These findings support the broader consensus that 60%–65% moisture provides the best balance of fibrous texture and sensory acceptability in HMMAs.

##### 3.1.4.3 Effect of Injecting Gases

Supercritical fluid CO_2_‐assisted extrusion (SCFX), and nitrogen‐assisted extrusion (NX) are being used to modify the functional and structural properties of proteins, particularly in the development of high‐protein extrudates (Alavi and Rizvi 2009; Lohani and Muthukumarappan 2017; Luo and Koksel 2023). Due to the ability of supercritical CO_2_ to dissolve within the matrix, SCFX enables controlled nucleation, allowing precise regulation of microcellular architecture in extruded products (Iqbal and Rizvi 2023). Furthermore, the low pH environment and precisely controlled temperature in SCFX help suppress Maillard browning, preserving the natural color, flavor, and nutritional integrity of extruded puffs while preventing excessive darkening and off‐flavor formation (Uhrin and Rizvi 2024). Recent studies have shown that SCFX enhances protein's hydration, emulsification, and structural properties by increasing water‐holding capacity, promoting protein denaturation, strengthening disulfide cross‐linking for fibrous texture, and improving hydrophobic interactions for network formation, making it highly suitable for meat analogs (Hosseini et al. 2024). In contrast, NX employs nitrogen gas as an expansion agent, incorporating it into the matrix through a whipping action that promotes aeration and porosity in the final extruded product (Luo et al. 2020). Studies on nitrogen‐assisted microfoaming in soy protein and wheat flour extrudates have shown that NX significantly improves protein emulsification, expansion, and crispiness while maintaining nutritional integrity (Li et al. 2019b). NX has also been applied in the production of puffed yellow pea and lentil‐based snacks, where nitrogen injection led to enhanced aeration and a well‐structured porous matrix, improving mouthfeel and product stability (Luo and Koksel 2023). Beyond conventional HME, steam‐based extrusion offers an alternative approach by utilizing high temperatures and water vapor to drive expansion. This method utilizes high temperatures and the expansive force of water vapor, generated during its phase change, to facilitate the expansion of the product. Unlike methods that use dry heat or other gases, steam‐based extrusion ensures more uniform heat distribution and enables faster cooking and texturizing, making it particularly effective for certain types of food products. These advancements highlight the potential of gas‐assisted extrusion in designing sustainable, high‐protein foods with enhanced structural and functional properties, paving the way for innovations in plant‐based proteins, high‐protein crisps, and nutrient‐rich extruded snacks.

##### 3.1.4.4 Effect of Environmental Factors on Fibrous Texture Stability

The stability and texture of PBMAs is influenced by various environmental factors, with storage conditions—particularly temperature and humidity—playing a pivotal role. These factors can impact the physical structure, moisture content, and protein interactions in the product, ultimately affecting its textural integrity and sensory appeal. Maintaining optimal storage conditions is critical to preserving the fibrous texture and ensuring product quality over its shelf life (Schmid et al. 2022). Temperature and humidity are primary factors influencing the stability and texture of PBMAs. Fluctuating temperatures can lead to structural changes in the protein matrix. High temperatures may accelerate the degradation of protein networks and the breakdown of noncovalent bonds such as hydrogen bonds and ionic interactions, leading to a loss of fibrous integrity (Choi et al. 2022). Conversely, freezing temperatures can result in ice crystal formation, which disrupts the protein matrix and causes textural softening upon thawing. Maintaining stable refrigeration (typically between 0 and 4°C) minimizes these risks, preserving the product's texture and sensory attributes (Dekkers et al. 2018). Humidity levels during storage also have a significant impact. High humidity causes moisture absorption, leading to product softening and altering its intended mouthfeel. On the other hand, low humidity can result in hardening or dryness of the texture (Choi et al. 2022). These changes not only affect the sensory qualities of the product but also its structural stability. Packaging innovations, such as vacuum sealing or modified atmosphere packaging (MAP), are often employed to mitigate these effects by controlling the product's exposure to environmental humidity (Ribeiro et al. 2024). While extrusion conditions are often optimized empirically, future progress requires a deeper coupling of rheological profiling with in‐line monitoring tools to predict and control fibrous structure development in real time. In our view, the influence of die geometry and cooling rate on the orientation and consolidation of protein phases remains underexplored, despite their decisive role in defining the final texture of PBMA.

### 3.2 Emerging Technologies for Fibrous Texture Formation

#### 3.2.1 3D Printing

3D printing, or additive manufacturing, is a rapidly emerging technology for creating complex fibrous structures in plant‐based proteins. Unlike traditional extrusion methods, 3D printing offers unprecedented precision in fabricating hierarchical structures, enabling the development of meat analogs that closely replicate the intricate textures of animal muscle (Dankar et al. 2018). The process involves layer‐by‐layer deposition of plant‐based protein materials, guided by computer‐aided design (CAD) models (Rayna and Striukova 2016). This technique allows for customizable shapes, textures, and even nutritional profiles in plant‐based food products. Proteins such as soy, pea, and WG are commonly used, often in combination with functional additives such as hydrocolloids and emulsifiers to improve the printability and texture of the end product. These additives enhance the flow behavior during printing and stabilize the deposited layers, ensuring a cohesive fibrous network upon solidification (Vallikkadan et al. 2023). Advances in 3D printing technology have enabled the construction of anisotropic fibrous structures that mimic the texture and mouthfeel of meat. By strategically blending proteins with starches or oils, researchers have achieved better alignment of protein molecules during printing (Dankar et al. 2018). This alignment results in hierarchical fiber assemblies that emulate the layered and fibrous structure of muscle tissue. Studies on plant‐based fish analogs have demonstrated how blending PP with corn starch and flaxseed oil enhances both printability and postprinting mechanical properties, leading to realistic fish fillet simulants (Wu et al. 2024). Despite its potential, 3D printing faces several challenges such as achieving high‐resolution printing with strong interlayer adhesion, which is difficult due to the complex interactions between protein molecules during and after deposition (Sohel et al. 2025). Additionally, the mechanical strength of 3D‐printed structures often falls short of traditional extrusion methods. To address these challenges, researchers are exploring enzyme‐assisted printing approaches, such as incorporating transglutaminase and laccase, to improve cross‐linking between protein molecules and enhance structural stability (Cheng et al. 2025).

#### 3.2.2 Shear Cell Technology

Shear cell technology is a method that applies controlled shear forces to align and restructure protein matrices, creating textures that closely mimic the fibrous nature of meat. By carefully managing the magnitude, direction, and duration of these forces, proteins can be oriented in specific patterns to replicate the alignment of muscle fibers found in animal meats (Cornet et al. 2022). This process involves heating and shearing protein solutions or dispersions within a specific shear cell apparatus, which aligns the protein molecules into layers or fibers. The textural properties achieved through this technique are particularly effective for simulating whole‐muscle cuts, such as chicken breasts or beef steaks. The simplicity and efficiency of shear cell technology make it a viable option for scaling up production while maintaining texture consistency (Wang et al. 2023b). It is also versatile, allowing for the use of various plant‐based protein sources, including soy, pea, and WG. Recent research highlights the potential of shear cell technology to produce products with low‐fat content, appealing to health‐conscious consumers while maintaining desirable sensory attributes (Cornet et al. 2022). A study by Dinani et al. (2023) demonstrated that l‐cysteine (CYS) and l‐ascorbic acid (AA) enhance the textural properties of PPI and WG blends in high‐temperature shear cell processing, with CYS improving tensile strength and AA inducing porosity and fibrous structures, aiding in better meat analog development. Couette Cell, a process that measures a fluid's rheological properties by applying shear between two concentric cylinders, where one rotates and the other remains stationary, can produce soy‐based meat analogs with fibrous structures under mild conditions, achieving optimal anisotropy at 120°C, 30 ± 5 min, and 25 ± 5 rpm. The upscaled system enables the production of large, meat‐like structures, such as emulating chicken breast or beef, with potential for industrial application (Krintiras et al. 2016).

#### 3.2.3 Electrospinning

Electrospinning is a cutting‐edge technique originally developed in the general domain of material science. It is now being adapted for food applications, particularly in plant‐based meat production. The process uses a high‐voltage electric field to draw ultrafine fibers from a liquid protein polymer solution. The electrospinning process utilizes a high‐voltage electric field (typically 10–30 kV) to draw charged protein polymer solutions into continuous, ultrafine fibers (diameter ranging from nanometers to micrometers), creating aligned fibrous structures that can mimic meat texture (Aghababaei et al. 2024a). These fibers are then collected to form intricate fibrous networks that replicate the fine, anisotropic structure of muscle tissue (da Trindade et al. 2024a). The fine control over fiber diameter and alignment in electrospinning makes it uniquely suited to produce textures that are both tender and complex, suitable for replicating delicate meats like chicken, fish, or shellfish. Electrospinning also enables the incorporation of other functional ingredients, such as lipids or flavors, directly into the fibers, enhancing the taste and sensory profile of the final product (Coelho et al. 2021). Da Trindade et al. (2024b) demonstrated the feasibility of electrospinning polysaccharides, zein, and poly (ethylene oxide) (PEO) to create fibers for PBMA, with the optimal 90:10 zein/carrageenan ratio providing hydrophilic surfaces, excellent moisture retention, and thermal stability, crucial for achieving consistent textures. Electrospun fibers were made using zein, PEO, and PP to enhance the sensory qualities of plant‐based meats. Zein/PEO blends improve hydrophilicity and thermal stability, while incorporating PP into a zein/PEO blend (20:1) further enhances fiber properties (da Trindade et al. 2024a). These fibers mimic the texture and appearance of animal meat, offering a promising solution for creating plant‐based meats with improved sensory resemblance. MacQueen et al. (2019) developed scalable microfibrous gelatin scaffolds for cultured meat production, replicating structural features of natural muscle tissue. Using immersion rotary jet spinning, gelatin fibers were produced at high rates with tunable diameters (1.3–8.7 µm) comparable to natural collagen fibers. Cross‐linked gelatin scaffolds supported the attachment, proliferation, and alignment of bovine and rabbit muscle cells, promoting structural and mechanical properties akin to meat, though lacking mature contractile architecture. Challenges remain in scaling this technique for industrial applications due to its complexity and reliance on high‐energy inputs. Recent advances in process efficiency and equipment design aimed at addressing these limitations are making electrospinning a promising avenue for future development. Table 1 summarizes the principles of key PBMA production techniques, including HME, 3D printing, shear cell technology, electrospinning, and others. The table highlights their relative advantages and limitations, providing a clearer understanding of their applications and challenges in plant‐based meat production. Although emerging technologies have demonstrated strong potential for generating aligned structures at the lab scale, a critical evaluation of their scalability and energy efficiency is required before they can be considered as viable complements to extrusion in industrial PBMA production. These technologies offer promising avenues for achieving structural precision and customization; however, their limited throughput and need for material standardization present significant barriers to commercial adoption.

**TABLE 1:** Comparative overview of key techniques for plant‐based meat analog production.

Technique	Principle	Advantages	Limitations	References
High‐moisture extrusion (HME)	Aligns plant proteins using heat, pressure, and shear under high‐moisture conditions in a twin‐screw extruder	‐Nutritional and structural benefits (e.g., enhanced digestibility, fibrous texture) ‐Versatility in incorporating diverse ingredients (plant and insect proteins) ‐Potential to address sustainability goals	‐High energy input; ‐Limited shape control due to die design and laminar flow ‐Knowledge gaps in underlying texturization mechanisms	Dekkers et al. (2018), Van Der Sman and Van Der Goot (2023), Zhang et al. (2023), Zhang et al. (2024a)
3D printing	Layer‐by‐layer deposition of a protein‐based paste, typically through extrusion‐based techniques, to construct defined shapes	‐Enhanced scalability ‐Reduced ingredient waste ‐ Ability to customize for diverse dietary and nutritional needs	‐Technical limitations in replicating the fibrous texture of real meat ‐High equipment and material costs pose economic barriers ‐ Slower production speed compared to conventional processing	Dong et al. (2023), Reuben et al. (2025)
Shear cell technology	‐Uses mechanical shear in a conical shear cell to align and structure proteins, promoting fibrous texture	‐Ability to produce large whole cuts of plant‐based meat (e.g., vegetarian steaks, pork fillets) without binders ‐ Produces long fibers with good texture and bite	‐High resource demand and scalability limitations ‐ As a batch process, it may struggle to maintain uniformity and efficiency at industrial scale	Cornet et al. (2022), De Angelis et al. (2024), Dekkers et al. (2018), Kumar et al. (2022)
Electrospinning	Uses high‐voltage electric fields to draw ultrafine fibers from a viscous protein solution, mimicking fibrous muscle structure	‐Fine fiber control ‐High product quality due to low contamination risk ‐Material flexibility ‐Environmental efficiency	‐Technical challenges in achieving desired fibrous texture with some plant proteins ‐Difficulty in scaling up ‐Use of solvents raises safety concerns	Cheng et al. (2024), Gagaoua et al. (2022), Topuz and Uyar (2024), Yu et al. (2023)
Wet spinning	Extrudes a polymer solution through a spinneret into a nonsolvent bath to induce phase separation and fiber solidification	‐High fiber quality with strong tensile properties and flexibility ‐Better mimicry of animal protein texture ‐Suitable for precise structural control	‐High energy and solvent use ‐Poor scalability. ‐Challenges in fat integration for marbled meat analogs	Cheng et al. (2024), Dinali et al. (2024), Yu et al. (2023)
Freeze structuring	Freezing of protein emulsions to create distinct fibrous structures that mimic the texture of animal meat. Controlled removal of heat facilitates ice crystal formation within the protein mixture	‐Produces satisfying juiciness and chewiness in meat analogs ‐ Aligns with sustainability goals	‐Energy‐intensive process raising environmental concerns and limiting commercial scalability ‐ Requires precise control over freezing parameters, leading to potential inconsistencies in product quality	Dekkers et al. (2018), Munialo et al. (2025)

## 4 Functional Ingredients and Structural Engineering of PBMA

The formation of fibrous textures in PBMA is a complex process influenced by multiple factors, including protein source and composition, process parameters, and the incorporation of functional additives. Each of these factors plays a crucial role in achieving the meat‐like structure that consumers expect. Some of the major factors affecting the fibrous structure formation are discussed below.

### 4.1 Protein Source

Plant‐based proteins are integral to the creation of meat analogs, particularly due to their unique functionalities, such as water absorption, emulsification, gelation, and texturization (Kothuri et al. 2025). The success of plant‐based formulations relies on the type, concentration, and purity of the protein source, which collectively determine the ability to replicate the fibrous texture of animal muscle. Since different plant proteins possess distinct structural properties, their selection is important in achieving the desired texture and sensory attributes in meat alternatives.

#### 4.1.1 Soy Protein

Soybean is an easily available and extensively used ingredient in meat analogs worldwide (Ishaq et al. 2022). It is available in three primary forms, including soy flour, soy protein concentrates (SPCs), and SPIs with approximate protein contents of 50%, 70%, and 90%, respectively (Webb et al. 2023). The extractable soy proteins are classified into four protein categories: 2S, 7S (β‐conglycinin, around 40% of total protein), 11S (glycinin, around 30% of total protein), and 15S according to their sedimentation coefficients. β‐Conglycinin (equivalent to vicilin in other legumes) and glycinin (equivalent to legumin in other legumes) fractions represent around 80% of the soy proteins (Kyriakopoulou et al. 2021). The functional properties of soy ingredients are determined by the type and ratios between different proteins and the presence of additional compounds such as carbohydrates (Kyriakopoulou et al. 2021). Both vicilin and legumin are salt‐soluble. Vicilin, also referred to as 7S globulin, is a trimeric protein typically composed of three subunits, each with a molecular weight ranging from 50 to 70 kDa, resulting in a native trimer complex of approximately 150–210 kDa (Qi et al. 2011). It is relatively heat‐labile, less prone to forming disulfide bonds, and has lower cysteine and methionine content, which limits its structural rigidity and network‐forming ability. Legumin, also known as 11S globulin, forms a hexameric complex made up of six subunit pairs (acidic and basic chains) linked via disulfide bonds. Each subunit pair is ∼60 kDa, yielding a total complex of around 320–360 kDa (Staswick et al. 1984). Legumin is more heat‐stable, has a higher sulfur amino acid content, and is capable of forming intermolecular disulfide bonds, contributing to stronger gelation and fibrous structuring under thermal processing. Both proteins exhibit pH‐dependent solubility, with isoelectric points (pI) in the range of 4.5–5.0, and show limited solubility near their pI (Renkema et al. 2001). However, legumin is a smaller fraction of the protein content in soy; it contains about 20 sulfide groups, allowing for disulfide bonding and a strong ability for texturization. Therefore, the ratio of these proteins is very important to the protein functionality in texturized vegetable proteins (Kyriakopoulou et al. 2021). Thus, despite the lower concentration of legumin compared to vicilin in soy protein, it plays a major role in the texturization of protein via a greater number of sulfide groups. Soybean protein has been designated the maximum possible score of 1.0 for being identical to animal protein on the scale of protein digestibility corrected amino acid score (PDCAAS; Ishaq et al. 2022). Owing to good nutritional composition and the ability to regulate textural properties of the final product, and satisfactory functional qualities such as the ability to absorb water and oil and its emulsifying properties, soy proteins are one of the most widely used and researched components of PBMA (Benković et al. 2023; Ishaq et al. 2022). Due to the excellent features of soy, it is often used as a standard or benchmark to compare different protein materials (Zahari et al. 2022). Recent advancements in PBMAs have focused on improving the textural and functional properties of SPC‐based high‐moisture meat analogs (SPC‐HMMA). A novel study by Gulzar et al. (2025b) integrated artificial intelligence (AI) and genetic algorithms (GA) to optimize the texture of SPC‐HMMA, aiming to mimic the characteristics of traditional chicken and beef. By adjusting sorbitol concentrations and moisture levels, the texture of SPC‐HMMA was significantly modified.

#### 4.1.2 Pea Protein

Peas contain 20%–30% protein, 50%–70% carbohydrates, and fiber, with composition varying by genotype and environment (Lu et al. 2020). They are a rich source of lysine and threonine, essential for human nutrition (Johansson et al. 2021), and provide minerals (Fe, P, Mg), vitamins, and lipids, enhancing their nutritional value (Benković et al. 2023). PP comprises four protein classes including globulin (55%–65%), albumin (18%–25%), prolamin (4%–5%), and glutelin (3%–4%; Lu et al. 2020). Within the globulin fraction, the primary proteins are vicilin (43%), a 7S globulin that contributes to foaming and emulsifying properties, and legumin (28%), an 11S globulin that enhances gelation and textural strength. Peas also contain convicilin, unique to this legume, which has a higher sulfur content than vicilin, aiding in disulfide bond formation crucial for textured plant proteins (Webb et al. 2023). PP exhibits several technofunctional properties, such as water‐ and fat‐binding abilities, making it an effective binder in meat analogs. It has moderate gelling capacity, though inferior to soy, and good emulsifying and foam‐stabilizing properties, making it versatile in food applications (Lam et al. 2018; Lu et al. 2020). High water‐holding capacity and solubility allow PP to form emulsions and protein networks, crucial for developing meat analogs (Lee et al. 2023). PP has been successfully structured using HME and shearing to create meat analog fibers (Osen et al. 2014; Schreuders et al. 2019). However, pea‐based structures are weaker than soy‐based ones, necessitating the use of hydrocolloids or structural agents to improve textural properties (Batista et al. 2005).

#### 4.1.3 Wheat Gluten

WG is composed primarily of two protein fractions: gliadin, the major prolamin, and glutenin, the major glutelin. Gliadins are monomeric proteins with molecular weights of 30,000–60,000, characterized by their ability to form intrachain disulfide bonds and impart viscosity to gluten (Peng et al. 2023; Veraverbeke and Delcour 2002). Glutenins are high molecular weight (up to 20 million) polymeric proteins that form interchain disulfide bonds, contributing to the elasticity and strength of gluten (Gianibelli et al. 2001; Peng et al. 2023; Sissons et al. 2005). Together, gliadin and glutenin form a cohesive, viscoelastic matrix when mixed with water, critical for both bakery, and meat analog applications (Webb et al. 2023). Cross‐linking via disulfide bonds between these proteins, particularly under heating and mechanical deformation, results in the formation of a robust 3D protein network (Peng et al. 2023). Gluten's ability to form extensible and elastic films under deformation makes it ideal for producing fibrous textures in plant‐based meat products (Sissons et al. 2005). Its water retention and swelling properties enhance cooking stability and slicing characteristics, reducing cooking losses and ensuring a consistent texture (Peng et al. 2023). Gluten contributes to the swelling and cohesiveness of the protein matrix, enabling it to be stretched and aligned under shear flow in the cooling die during extrusion. This behavior helps mimic the fibrous texture of muscle meat in PBMA (Krintiras et al. 2015). HME processes utilize gluten's viscoelastic properties to transform protein matrices into fibrous structures. This is achieved through mechanical stretching and deformation, which aligns the protein molecules to produce anisotropic textures similar to real meat (Krintiras et al. 2015). The polymerization of gluten proteins under extrusion conditions, driven by disulfide bond formation and rearrangement, creates a continuous network essential for meat‐like textures (Kyriakopoulou et al. 2021). Gluten also functions as a binder, ensuring that the fibers are held together in the final product, contributing to the chewiness and elasticity desired in plant‐based meat (Kumar et al. 2017; Zhang et al. 2021).

Factors like temperature, water content, and shear force significantly influence gluten polymerization and fibrous structure development. Extrusion temperatures between 130 and 140°C promote anisotropic structures by enhancing protein alignment and cross‐linking, while temperatures above 140°C may lead to degradation (Emin et al. 2017). Lower temperatures (< 80–90°C) result in weaker network formation, with reduced polymerization and fibrous characteristics, leading to more isotropic textures (Emin et al. 2017). Optimal water content (20%–40%) enhances gluten's viscoelastic properties, promoting fiber formation and mechanical strength. Water levels below 20% lead to dense, brittle structures due to restricted protein mobility, while exceeding 40% reduces cross‐linking efficiency, resulting in a weaker gluten network (Emin et al. 2017). Shear forces during extrusion promote fibrillation and fiber orientation, enhancing meat‐like textures. Optimal shear conditions (∼150 rpm screw speed) result in the best fibrous structure, whereas excessive shear (> 200 rpm) can degrade the network, leading to a gel‐like texture instead of meat‐like fibers (Zhang et al. 2022). Low lipid concentrations (< 2%) act as plasticizers, moderating protein aggregation and enhancing hydrophobic interactions, which helps maintain gluten elasticity and flexibility. However, lipids also reduce excessive cross‐linking, preventing a dense, overly rigid structure, thereby influencing the final texture (Chen et al. 2021). When combined with soy protein, WG enhances fibrous structure formation by acting as the continuous phase in a two‐phase system. The interactions between gluten and soy protein influence water distribution and texture development, but structural integrity depends on processing conditions (Peng et al. 2023).

#### 4.1.4 Rice Protein

Rice protein can be derived from defatted rice bran, a byproduct of rice milling, which contains 15.4% protein (Amagliani et al. 2017). Rice protein primarily comprises glutelins (80%), followed by prolamins (4%), globulins (12%), and albumins (2%–4%) (47). Glutelins are hydrophobic and held together by disulfide bonds, making them structurally stable in acidic or alkaline solutions (Amagliani et al. 2017). Rice protein is hypoallergenic, making it ideal for sensitive populations such as infants and the elderly (Zheng et al. 2023). It also boasts a high biological value (77%) compared to soy protein (67%) and comparable to shrimp protein (Li et al. 2019b). Rice protein is considered superior to proteins from wheat and corn and comparable to oats, with an amino acid composition that surpasses casein and SPI (Han et al. 2015). The functional properties of rice protein, such as solubility, foaming, emulsifying, gelling, water‐holding, and oil‐holding capacities, are critical for its food applications. However, its poor solubility negatively affects these properties (Amagliani et al. 2021). Several modification methods, such as chemical methods, enzymatic methods, physical methods, have been explored to improve solubility. Despite these advances, combining rice protein with other protein systems may yield better functional outcomes (Zheng et al. 2023). Technological advancements have enabled rice protein to be incorporated into meat analogs, enhancing their nutritional value. Combining rice protein isolate with SPI has produced improved meat analogs. Qiu et al. (2023) reported that incorporating rice protein enhances 3D printing capabilities for creating meat‐like shapes and textures. Unlike soy protein, rice protein has a milder flavor, lacks an unpleasant taste, and is both hypoallergenic and hypocholesterolemic (Detchewa et al. 2022). It supplements methionine, an amino acid often deficient in soy protein, while lysine, the limiting amino acid in rice protein, is abundant in soy protein, making it nutritionally complementary. Consuming these proteins together can help in preventing amino acid deficiencies (Lee et al. 2022b). Rice protein has a lower water‐holding capacity compared to soy protein, about one‐third of that of soy protein. Despite this limitation, rice protein has potential as an ingredient for meat analogs (Lee et al. 2022a). The integration of rice protein into meat analogs is a promising yet underexplored area. Its nutritional and functional properties, particularly when combined with soy protein, make it a viable alternative for producing meat analogs with improved amino acid profiles. Further research and technological innovations could unlock their full potential, addressing challenges related to solubility and industrial scalability.

#### 4.1.5 Other Sources

Lentil proteins are emerging as a promising ingredient in plant‐based meat alternatives due to their excellent nutritional value, functional properties, and sustainability (Boye et al. 2010). Lentil proteins enhance the nutritional profile of plant‐based formulations, especially when combined with other sources like pea or rice protein to achieve a complete amino acid profile (Boye et al. 2012). Lentil proteins exhibit desirable functional properties, including high water‐holding capacity, gelation ability, and emulsification properties, which are critical for mimicking the fibrous, juicy texture of meat (Karaca et al. 2011). During extrusion and shear cell processing, lentil proteins can form cohesive, anisotropic structures that replicate the texture of whole‐muscle cuts, such as chicken or fish (Dekkers et al. 2018). Although challenges like solubility and flavor masking remain, advances in processing technologies and ingredient engineering are unlocking the potential of lentil proteins as a versatile and eco‐friendly alternative in the growing market for PBMA (Asgar et al. 2010). The technofunctional properties of lentil proteins include a solubility range that varies depending on processing conditions. Solubility is highest under neutral to alkaline conditions, reaching 55%–65% at pH 7.0 and improving further at pH 9. However, it is significantly reduced at pH 4–5, near the isoelectric point, leading to protein aggregation and precipitation (Aghababaei et al. 2024b). Lentil proteins exhibit an emulsification activity index (EAI) between 90.3 and 123.3 m^2^/g, with an average value of 93.3 ± 0.2 m^2^/g, depending on processing conditions. These properties contribute to their role in stabilizing emulsions for plant‐based formulations (Joshi et al. 2011). Lentil proteins exhibit a water absorption capacity ranging from 2.8 to 4.1 g/g protein and an oil absorption capacity of 1.6–2.9 g/g protein, depending on processing conditions and protein fractionation methods. These functional properties influence texture and mouthfeel in plant‐based formulations (Chang et al. 2023). Lentil proteins require relatively low concentrations (8%–14% w/v) to form gels upon heating, indicating stronger gelling ability compared to pea, chickpea, and soy proteins. (Jarpa‐Parra et al. 2014). While these concentrations are lower than those typically required for PBMAs, this property makes lentil proteins advantageous as thickening ingredients to modulate food texture at lower protein levels.

Chickpea proteins are increasingly recognized as a versatile and nutritionally rich option for plant‐based foods (Boye et al. 2010). With a protein content ranging from 17% to 22% in raw chickpeas and higher concentrations in isolated forms, chickpea proteins offer excellent digestibility and a favorable balance of essential amino acids, particularly lysine (Boukid 2021). These properties make them a valuable ingredient for complementing other plant proteins, such as wheat or pea, in achieving a complete amino acid profile in meat substitutes (Day 2013). Chickpea proteins excel in their emulsification (50–75 m^2^/g), water‐binding (2.9–4.5 g/g protein), and gelation (12%–16% w/v) properties, making them essential for cohesive textures in plant‐based formulations (Pennells et al. 2024). Their emulsifying capacity stabilizes fat and water mixtures, contributing to the juicy and tender textures often associated with traditional meat products (Makri et al. 2005). Chickpea protein gels are heat‐induced, forming stable networks that mimic the fibrous structure of meat, especially when processed via HME or shear cell technology (Dekkers et al. 2018). These attributes make chickpea proteins suitable for applications in burgers, nuggets, and even whole‐muscle analogs (Boukid 2021). One notable advantage of chickpea proteins is their mild flavor profile compared to other plant proteins, such as soy or pea. This characteristic reduces the need for extensive flavor masking, making chickpea proteins ideal for clean‐label formulations (Boye et al. 2010). Beyond their functional roles, chickpeas are hypoallergenic and non‐GMO, broadening their appeal to health‐conscious and allergen‐sensitive consumers (Boukid 2021). From a sustainability perspective, chickpeas are a low‐impact crop with moderate water requirements and the ability to fix nitrogen, contributing to soil health (Nemecek et al. 2008). These attributes position chickpea proteins as an environmentally friendly alternative to traditional meat and other plant protein sources (Tharanathan and Mahadevamma 2003). Despite their advantages, chickpea proteins can exhibit lower solubility under certain conditions, which may require optimization through advanced extraction and processing methods (Joshi et al. 2011). Their application in PBMA is expanding, driven by innovations in protein structuring technologies and their growing consumer demand for sustainable, nutrient‐rich options (Dekkers et al. 2018). Boukid (2021) highlighted chickpea proteins as a promising alternative protein source, emphasizing their nutritional value, essential amino acid content, and digestibility. Their bland flavor, neutral taste, and light color, combined with functional properties, make them suitable for diverse food applications, including PBMA, noodles, breads, and sausages, with hydrolysates showing potential for broader use as functional ingredients (Boukid 2021). Zahari et al. (2023) found that HMMA made with hempseed protein concentrate (HPC) and WG at a 90:10 ratio showed superior textural properties, while 50:50 blends of HPC with WG or chickpea protein concentrate (CPC) provided balanced physicochemical and sensory qualities under optimized extrusion conditions.

Fava bean proteins are gaining recognition as a sustainable and functional ingredient for PBMA, offering a unique combination of nutritional benefits and versatile functional properties (Boye et al. 2010). With a protein content ranging between 25% and 30%, fava beans are an excellent source of essential amino acids, particularly lysine, which complements other plant proteins like WG or PP (Boye et al. 2010). Their high‐protein concentration makes FPIs and concentrates suitable for formulating meat analogs with balanced amino acid profiles (Alonso‐Miravalles and O'Mahony 2018). Fava bean proteins excel in their water‐holding capacity (2–4 g/g protein) and emulsification properties, with an emulsifying activity index (45.6–67.8 m^2^/g) and emulsion stability index (55%–75%). These properties are critical for replicating the juiciness and cohesiveness of traditional meat products. During HME, fava bean proteins form heat‐induced gels and fibrous structures, closely mimicking the texture of muscle tissue (Dekkers et al. 2018). Their gelation ability enables the development of firm and elastic textures, making them suitable for a range of applications, including burgers, sausages, and whole‐muscle cuts (Huamaní‐Perales et al. 2024). Furthermore, enzymatic treatments can enhance the textural integrity of fava bean‐based products, improving their chewiness and mechanical strength (Eckert et al. 2019). Advancements in extrusion and shear cell technology, along with innovative ingredient engineering, are enhancing the use of fava bean proteins for improved texture, flavor, and nutrition (Dekkers et al. 2018). do Carmo et al. (2021) found that FPC can produce meat analogs with optimal firmness and elasticity under HME at 130–140°C, a water‐to‐feed ratio of 4, and a feed rate of 11 rpm, with moisture content being the most influential factor.

### 4.2 Amino Acid Profiles

The amino acid profiles of plant proteins play an important role in their ability to form fibrous structures, a key requirement in producing meat analogs. Specific amino acids contribute to protein alignment, bonding, and gelation, which are important for creating the fibrous anisotropic structures characteristic of meat. Glutamine and proline, found predominantly in WG, are crucial for forming hydrogen bonds that strengthen protein–protein interactions, providing elasticity and cohesion in fibrous structures (Varayil et al. 2024). Proline's cyclic structure provides rigidity and supports the alignment of protein strands, making WG an excellent choice for anisotropic fibrous structures in meat analogs (Wang et al. 2017). Lysine, abundant in soy protein, is essential for forming ionic and covalent bonds, enhancing protein cross‐linking, and improving texturization processes. It also improves the nutritional value of plant proteins, particularly in cereals where lysine is typically deficient (Dai et al. 2023). Sulfur‐containing amino acids, such as methionine and cysteine, are essential for forming disulfide bonds that contribute to structural rigidity and elasticity; however, these amino acids are often deficient in many plant proteins, necessitating supplementation or blending (Nishaant et al. 2025). Arginine supports cross‐linking and interactions with polysaccharides, improving gelation and overall matrix strength, which is critical for fibrous texture development (Varayil et al. 2024). Branched‐chain amino acids such as leucine, isoleucine, and valine promote hydrophobic interactions, aligning protein chains during processing and improving resilience under mechanical stress. The fibrous texture of meat analogs is achieved through the alignment of protein molecules into anisotropic structures via hydrogen bonding (glutamine), hydrophobic interactions (branched‐chain amino acids), and disulfide bond formation (cysteine), particularly during extrusion (Nishaant et al. 2025). WG, with its high glutamine and proline content, forms strong elastic networks, which are essential for creating the chewiness of meat analogs (Chiang et al. 2021).

Soy protein, with a balanced amino acid profile, supports cross‐linking, gelation, and emulsification, making it versatile for mimicking meat‐like textures and mouthfeel (Ribeiro et al. 2024). Protein blending, such as combining pea and rice protein, compensates for amino acid deficiencies and ensures the creation of a complete protein matrix suitable for forming fibrous structures (Maningat et al. 2022). Challenges in meat analog production include the amino acid imbalance in many plant proteins, such as deficiencies in cysteine, methionine, and lysine, necessitating fortification or blending (Nishaant et al. 2025). Overprocessing risks denaturing proteins, reducing their ability to form fibrous structures, while achieving consumer acceptance requires textures that closely mimic meat through precise protein composition and processing (Varayil et al. 2024). The final products must replicate meat‐like characteristics, including chewiness and juiciness, through proper amino acid balancing and optimized processing methods (Jang and Lee 2024). By engineering amino acid profiles and employing advanced processing techniques, manufacturers can create high‐quality fibrous meat analogs that meet functional, structural, and nutritional demands (Varayil et al. 2024).

### 4.3 Functional Properties of Proteins

The functional properties of plant proteins are important in creating fibrous structures that replicate the sensory and structural qualities of animal‐derived meat. The functional properties, such as water absorption, oil absorption, gelling capability, elasticity, and viscosity, influence the textural attributes and ensure the structural integrity and palatability of meat analogs. Water absorption is fundamental to texture formation as it allows plant proteins to hydrate and swell, enabling them to interact effectively with other ingredients (Flory and Alavi 2024). During HME, this hydration facilitates the alignment of protein molecules into anisotropic fibrous structures, contributing to elasticity and firmness that mimic muscle fibers (Kyriakopoulou et al. 2021). This capacity to retain moisture is essential for maintaining the juiciness and structural stability of meat analogs, making it a pivotal property in replicating the mouthfeel of traditional meats (Flory et al. 2023).

Oil absorption enhances the juiciness and richness of meat analogs, contributing significantly to their flavor retention and sensory experience (Benković et al. 2023). The incorporation of oils within protein matrices during extrusion supports lubrication between fibers, improving texture and palatability (Benković et al. 2023). By enabling the fibrous network to hold fats, this property ensures that the final product replicates the succulence of animal meats (Younis et al. 2023). Gelling capability is another essential functional property, as it provides a cohesive matrix that holds water, oil, and other ingredients together (Benković et al. 2023). Proteins like soy and pea, which exhibit heat‐induced gelation, are instrumental in forming elastic and robust structures (Ge et al. 2023). The resulting gels interconnect protein fibers, producing a meat‐like firmness and fibrous texture that closely resembles muscle tissues (Webb et al. 2023). Some selected functional properties of plant proteins in the development of PBMA in different studies have been tabulated in Table 2.

**TABLE 2:** Selected functional properties of common plant proteins used for the development of PBMA.

	Functional properties			
Plant protein	Water‐holding capacity (g/g)	Oil‐binding capacity (g/g)	Least gelling concentration (% w/v)	References
Soy	3.5–5	2.5–3	12–7	de Paiva Gouvêa et al. (2023), Ma et al. (2022), Zhao et al. (2020)
Pea	2.5–4	1.5–2	8–15	de Paiva Gouvêa et al. (2023), Zhao et al. (2020)
Wheat gluten	1.5–2.5	1–1.5	6–8	Zhao et al. (2020)
Rice	1–2	0.5–1	12–15	Zhao et al. (2020)
Lentil	2.5–3.5	1.8–2.5	8–14	Boye et al. (2010), Joshi et al. (2011), Ma et al. (2022)
Chickpea	2–3	1.5–2	10–13	Boye et al. (2010)
Fava	2–4	2–2.5	8–12	de Paiva Gouvêa et al. (2023), Ma et al. (2022)

Elasticity and viscosity further enhance the structural and sensory attributes of meat analogs (Schreuders et al. 2021). Elasticity allows proteins to stretch and recover during extrusion, enabling the formation of long, fibrous strands (Zhang et al. 2022). Simultaneously, viscosity helps maintain cohesiveness and alignment under mechanical stress, ensuring the continuous formation of fibers without breakage (Kyriakopoulou et al. 2021). These properties are crucial for replicating the chewiness and fibrous nature of muscle meat, which are central to the sensory appeal of PBMA (Flory et al. 2023). These functional properties work synergistically during processing, particularly in HME, where heat, pressure, and mechanical shear align protein molecules into fibrous forms (Benković et al. 2023). By optimizing the balance of water absorption, oil retention, gelation, elasticity, and viscosity, manufacturers can create high‐quality, fibrous meat analogs that meet not only functional and structural demands but also provide nutritional adequacy (Webb et al. 2023). This comprehensive approach ensures that PBMA delivers a sensory experience comparable to that of animal meat.

### 4.4 Additives in Fibrous Texture Formation

The use of additives in formulations is a critical aspect of developing PBMAs, as these ingredients enhance the structural, sensory, and functional properties of the final product. Additives such as binding agents, fat mimetics, and flavor enhancers play complementary roles in improving the fibrous texture, juiciness, and overall acceptability of plant‐based proteins. The effectiveness of texturization depends on the rheological properties of the protein dough, which must possess sufficient elasticity and plasticity to withstand mechanical manipulation without tearing or breaking (Jang and Lee 2024). The inclusion of hydrocolloids, such as xanthan gum or guar gum, can improve dough elasticity, facilitating more effective stretching and alignment (Baig et al. 2025). Additionally, protein blends, such as soy and WG, exhibit enhanced mechanical properties, making them particularly suitable for texturization techniques (Ribeiro et al. 2024). Binding agents, such as methylcellulose, xanthan gum, guar gum, and konjac glucomannan, play a pivotal role in forming the protein matrix and improving water retention (Yekta et al. 2023). These hydrocolloids interact with proteins and water to create a cohesive network that mimics the elasticity and chewiness of meat. Methylcellulose undergoes gelation upon heating, which helps stabilize the protein structure during cooking, resulting in a firm yet tender texture (Steinke 2001). Similarly, xanthan gum enhances viscosity and elastic properties, allowing the protein matrix to hold its shape under mechanical stress or during cooking (Sandhu et al. 2015). Hydrocolloids also improve the processability of plant‐based formulations, ensuring that the dough exhibits optimal rheological properties for extrusion, kneading, or other mechanical processing techniques. Their ability to bind water prevents excessive moisture loss during cooking, contributing to juicier textures and a more satisfying bite (Ribeiro et al. 2024).

Fats and oils are essential for replicating the juiciness and mouthfeel of animal meat. In PBMAs, plant‐based fats such as coconut oil, canola oil, or shea butter, as well as innovative fat mimetics, are incorporated to emulate these properties. Coconut oil is commonly used for its ability to solidify at room temperature and melt during cooking, providing a sensation similar to animal fat (Wang et al. 2015). Shea butter, with its smooth texture and mild flavor, is another popular choice (Lovett 2015). In recent years, structured emulsions and oleogels have gained attention as advanced fat mimetics (Li et al. 2022). These formulations combine plant oils with structuring agents to create solid‐like fat systems that behave similarly to animal fat in terms of texture and melting behavior. They are particularly effective in delivering a juicy, tender mouthfeel without contributing excessive greasiness (Li et al. 2022). Furthermore, these fat mimetics can be customized to include omega‐3 or omega‐6 fatty acids, enhancing the nutritional value of the product (Zhang et al. 2025). Recent studies have demonstrated that oleogels and emulsion gels can mimic intramuscular fat, improving both moisture retention and mouthfeel in PBMA (Tan et al. 2023).

**FIGURE 4:**
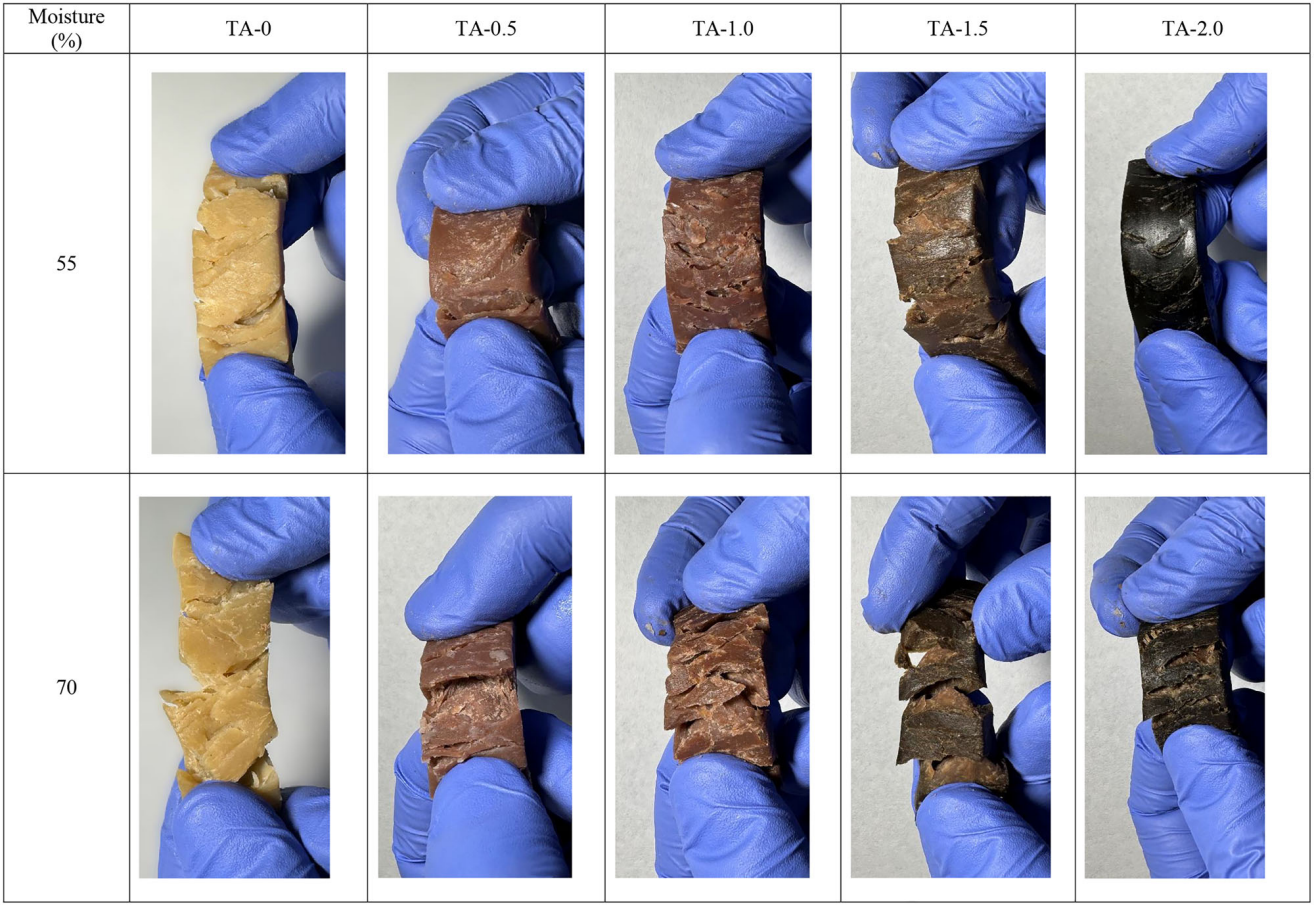
Photographs of soy protein concentrate‐based high‐moisture meat analogs (SPC‐HMMA) with different levels of tannic acid (TA) and target moisture contents. This figure illustrates the appearance of SPC‐HMMA and the effects on the macrostructure of HMMA with the addition of TA. Images adapted from Gulzar et al. (2025a).

The addition of polyphenols such as tannic acid can also affect the fibrous structure of the PBMA. In a study, the effects of tannic acid (TA) on the structural and functional properties of SPC‐HMMAs produced by extrusion were studied (Gulzar et al. 2025a). TA incorporation increased hardness and cutting strength, while moisture content influenced textural properties. Protein structural changes were observed through SEM and FTIR analysis. Technofunctional properties like cooking yield, water absorption, oil absorption, and antioxidant activity were significantly affected by TA. The images of SPC‐HMMA added with TA at different concentrations and different moisture levels are presented in Figure 4. In another study by Gulzar et al. (2025b), the impact of sorbitol addition on the texture of SPC‐HMMA was investigated. The findings revealed that sorbitol acted as a plasticizer, softening the texture of the HMMAs by reducing rigidity and enhancing flexibility within the protein matrix. Although flavor enhancers do not directly impact texture, they are crucial for creating a convincing sensory experience, as the perception of texture is closely tied to flavor. Ingredients like yeast extracts, amino acids, and natural flavor compounds derived from mushrooms or seaweed can enhance umami notes, which complement the texture of PBMAs (Nowacka et al. 2023). These enhancers also mask off‐flavors that are often present in plant proteins, such as the beany notes associated with soy or the grassy undertones of PP. By improving the overall flavor profile, flavor enhancers can positively influence how consumers perceive the texture, making it more palatable and closer to the experience of eating meat (Ribeiro et al. 2024).

**FIGURE 5:**
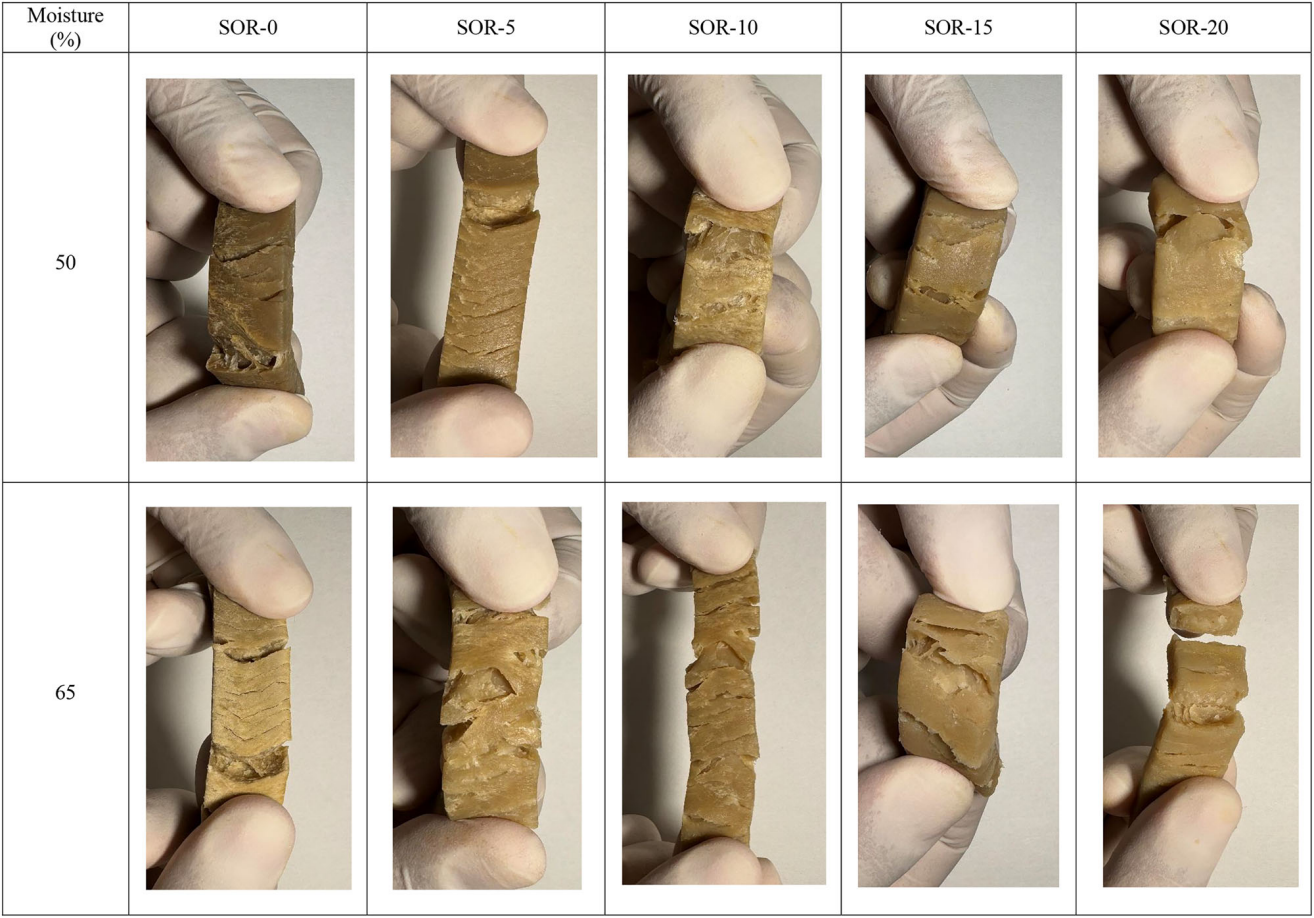
Pictures of soy protein concentrate‐based high‐moisture meat analogs (SPC‐HMMA) taken at different levels of sorbitol and target moisture content show variations in the textural properties of the HMMA. Images adapted from Gulzar et al. (2025b).

These sensory attributes are closely linked with food structure, influencing flavor perception, particularly through the release and retention of volatile compounds. HME processes provide a platform for enhancing these qualities through ingredient modifications and interactions within the extruder. The incorporation of yeast extract, a natural flavor enhancer derived from edible yeast, has been explored for its ability to impart meaty and umami flavors to PBMA (Mahadevan and Farmer 2006). The unique properties of yeast extract make it particularly suitable for use in HME, where its interaction with proteins during processing could enhance the flavor profile of PBMA. Polysaccharides, often added during extrusion, also contribute significantly to texture and moisture retention by interacting with proteins to form a stable matrix (van der Sman and van der Goot 2023). While these interactions improve structural homogeneity, they may also impact flavor perception, as the structural changes induced by polysaccharides influence the release and retention of volatile flavor compounds (Funami 2016). Plasticizers like water, glycerol, and lipids improve the texture and processability of PBMAs by increasing the flexibility and mobility of protein networks. Water, in particular, acts as both a hydration medium and a plasticizer, aiding in fiber formation during extrusion (Kyriakopoulou et al. 2021). The images of SPC‐HMMA containing sorbitol at different levels and varied moisture contents taken from a recent study are presented in Figure 5. The PBMA images reveal distinct textural changes in SPC‐HMMA with varying sorbitol concentrations and moisture levels. Without sorbitol (SOR‐0), the texture is dry and cracked, while 5%–15% sorbitol results in smoother, more elastic surfaces, with 15% producing the most homogeneous texture. At 20% sorbitol, the structure becomes overly soft and fragile. Similarly, increasing moisture from 50% to 65% softens and enhances fibrousness but leads to brittleness at higher levels. These images highlight the balance needed to optimize texture. Overall, we consider the strategic use of protein–polysaccharide complexes an underutilized opportunity to enhance water retention, juiciness, and structural stability in PBMA, particularly under HME conditions. Although compositional effects on extrusion behavior are well documented, their direct translation into sensory perception remains limited, highlighting the need to bridge molecular formulation with consumer‐oriented outcomes.

## 5 Protein Source Dynamics

Transitioning from animal‐based to plant‐based products presents unique challenges and opportunities in food technology. This section delves into the critical factors that must be addressed to successfully replicate the sensory and nutritional qualities of meat. Through a combination of innovative processing techniques and ingredient engineering, developers aim to create plant‐based alternatives that not only appeal to the palate but also meet dietary needs.

### 5.1 Flavor Profile

Flavor is one of the most complex and critical sensory attributes of meat and meat analogs, directly influencing consumer acceptance. Animal meats inherently possess compounds like peptides, amino acids, and lipids that undergo Maillard reactions during cooking, generating the characteristic umami‐rich and savory “meaty” flavors (Hossain et al. 2025). Plant proteins, in contrast, lack these flavor precursors and often require flavor additives and enhancers to achieve comparable profiles. The Maillard reaction, a chemical process between amino acids and reducing sugars, is central to the development of meaty flavors in animal proteins. Plant‐based proteins like soy and pea often have insufficient free amino acids and sugars, limiting their ability to generate similar compounds. Advances in formulation, such as fortifying plant‐based products with specific amino acids and sugars, have improved their ability to produce these desirable flavors during cooking (Contato and Conte‐Junior 2025). Additionally, fermentation techniques are being explored to enhance the flavor precursors in plant proteins, making them more suitable for flavor development.

One of the challenges with plant proteins is their inherent off‐flavors, often attributed to compounds like isoflavones, saponins, and alkaloids. These compounds impart bitter, beany, or grassy notes that can compromise the sensory appeal of PBMA. PP is known for its distinct earthy and bitter flavor, which requires masking or removal. Innovative methods such as supercritical CO_2_ extraction and ethanol washing have shown promise in reducing off‐flavors by removing these undesirable compounds (Iqbal et al. 2025). Moreover, enzymatic treatments and hydrocolloids have been employed to bind or neutralize off‐flavor molecules. PBMA often rely on flavor enhancers, such as yeast extracts, hydrolyzed vegetable proteins, and umami‐rich compounds like monosodium glutamate, to replicate the depth and complexity of meat flavors (Finlay‐Smits and Loeffen 2019). Yeast extracts can provide savory notes and mimic the umami characteristic of animal meat. Additionally, smoke flavors and spices are commonly used to mask off‐flavors and enhance the overall sensory experience. In animal meat, fat plays a dual role as a flavor reservoir and a medium for flavor release during cooking. Plant‐based products typically lack this functionality due to the absence of animal fat. Oleogels and structured emulsions are increasingly used in plant‐based formulations to replicate the flavor‐carrying properties of animal fats. These innovations allow for better retention and release of added flavors during cooking, enhancing the sensory profile of plant‐based analogs (Czapalay and Marangoni 2024; Tan et al. 2023). Despite advancements, the flavor profile of PBMA often remains a significant barrier to consumer acceptance. While efforts to replicate the umami and savory notes of animal meat have been partially successful, the nuances of flavor, such as depth, richness, and aftertaste, are still challenging to achieve. Future research is focusing on bioengineering and fermentation‐based flavor enhancement to create more authentic and appealing flavor profiles in plant‐based products.

### 5.2 Manufacturing and Sustainability

Plant‐ and animal‐based protein processing differ in sustainability, with plant‐based alternatives often seen as eco‐friendly. However, energy‐intensive steps like protein extraction, fractionation, and advanced structuring (HME, shear cell processing, 3D printing) are required to replicate meat‐like textures, potentially offsetting sustainability gains if not optimized. In contrast, animal meat production is less reliant on intricate processing but requires significant natural resources during livestock farming, including land, water, and feed, as well as high greenhouse gas emissions, particularly methane from ruminants. PBMA have a significantly lower environmental footprint compared to animal proteins. Producing plant‐based analogs uses less water, emits fewer greenhouse gases, and requires less agricultural land. These advantages make plant‐based alternatives a sustainable option for addressing global food security and mitigating climate change. However, challenges remain in ensuring the sustainability of raw material sourcing. The cultivation of protein crops like soy and wheat often relies on intensive monoculture farming, which can lead to soil degradation and biodiversity loss. Efforts are being made to diversify protein sources, such as incorporating fava beans, chickpeas, and lentils, which are less resource‐intensive and contribute to soil health through nitrogen fixation (Augustin and Cole 2022). Advanced processing technologies, while energy‐intensive, are being refined to improve efficiency and reduce environmental impact. Techniques like dry fractionation and cold extrusion require less water and energy compared to traditional wet processing methods, making them more sustainable options for protein isolation and texturization (Mandala and Apostolidis 2024). Additionally, integrating renewable energy sources into production facilities can further reduce the carbon footprint of PBMA manufacturing. The production of animal‐based proteins faces significant sustainability challenges, including high greenhouse gas emissions, land degradation, and water overuse. Livestock farming accounts for 16.5% of global greenhouse gas emissions, with beef production being a major contributor (Twine 2021). Reducing reliance on animal proteins through the adoption of plant‐based alternatives has the potential to alleviate these environmental pressures. The shift toward plant‐based alternatives also involves economic and social dimensions. While PBMA can offer long‐term cost savings through more efficient resource use, its production costs remain high due to the complexity of processing and the need for specialized equipment. Policies promoting investment in sustainable processing technologies and public awareness campaigns can support the broader adoption of plant‐based products.

### 5.3 Consumer Perception and Adaptability

The perception of PBMA significantly hinges on their ability to replicate the sensory and emotional experience provided by animal meat. Flavor, texture, and appearance are critical determinants for consumer acceptance, with animal meats naturally excelling in these areas due to their intrinsic properties. PBMA often faces challenges in matching these sensory qualities, necessitating advanced processing techniques and additives to bridge the gap (He et al. 2020). Animal meat provides a rich umami flavor, natural juiciness, and fibrous texture, which are inherently difficult to replicate in PBMA. Consumers often perceive PBMA as lacking in flavor complexity and textural fidelity. Innovations such as flavor enhancers, oleogels for fat mimicking, and HME have significantly improved the sensory profiles of PBMA. However, the emotional connection to traditional animal meats remains a barrier to wider acceptance, particularly among older consumers. While animal meat is viewed as a complete protein source, PBMA are increasingly fortified with vitamins and minerals to match the nutritional profile (Locatelli et al. 2025). Despite this, some consumers associate PBMA with overprocessing, which may dampen their perceived health benefits compared to the “natural” image of animal meat. Sustainability and ethics hold a distinct advantage in sustainability, resonating with environmentally conscious consumers. They generate fewer greenhouse gas emissions, require less water, and occupy less land compared to animal farming. This eco‐friendly aspect is a compelling driver for younger demographics, particularly millennials and Gen Z. However, older consumers often exhibit less willingness to transition from traditional meat due to established habits and skepticism toward alternative proteins. Animal diets are deeply rooted in cultural practices and traditions, making PBMA a less obvious choice for many consumers. Additionally, 3D printing and other cutting‐edge technologies allow for personalized PBMA formulations, catering to diverse consumer needs and preferences.

## 6 Future Research Directions

From the consumer's perspective, one of the most urgent needs in this field is to move beyond mimicking the visual and structural attributes of meat and instead focus on solving the deep‐rooted sensory and functional gaps that lead to consumer rejection. While technologies like HME have made significant progress, they often fail to deliver consistency across batches or match the dynamic oral processing characteristics of real meat. Future research must not only improve processing fidelity but also account for sociocultural, nutritional, and psychological dimensions of meat substitution. It is reasonable to believe that the industry may benefit from a strategic pivot—not just chasing meat imitation—but also exploring new categories of high‐protein, fiber‐rich, minimally processed plant‐based foods with their own sensory appeal. This could unlock new consumer segments and ease the technological burden of full mimicry. At the same time, continued advancements in technology to replicate the complex fibrous textures of meat remain important, particularly for consumers seeking close analogs to traditional meat products. Balancing both innovative paths will be key to meeting diverse consumer expectations. To support both approaches, developing meat analogs and creating novel plant‐based foods, advancements in processing technologies are essential. Emerging methods such as electrospinning, shear cell processing, and multimaterial 3D food printing offer promising solutions to overcome current limitations and expand the design space for future products. Further optimization of extrusion parameters and exploration of novel structuring agents, such as polysaccharides or cross‐linking enzymes, could lead to improved texture and mouthfeel. Research into combining these techniques with traditional extrusion could create multiscale textures that closely resemble natural muscle tissue. While plant‐based proteins provide an eco‐friendly alternative to animal proteins, their nutritional profile often lacks completeness. Future research should focus on blending complementary plant proteins, such as pea and rice, to achieve a balanced amino acid profile. Advances in bioengineering could enable the development of plant proteins with enhanced essential amino acid content or improved digestibility. Additionally, fortification with vitamins and minerals such as vitamin B12, heme iron, and zinc will be critical for ensuring that plant‐based analogs can meet specific nutritional targets typically associated with animal meat. At the same time, plant‐based formulations present opportunities to reduce components of concern in conventional meat, such as saturated fat and sodium, offering a potential health advantage. Innovations in microencapsulation technology may improve the stability and bioavailability of these nutrients. PBMAs are already recognized for its reduced environmental footprint compared to animal meat. However, further studies are needed to quantify their long‐term sustainability impact, particularly regarding raw material sourcing and processing efficiency. Exploring the potential of underutilized protein sources, such as algae or byproducts from other food industries, could enhance resource utilization and reduce environmental strain. Life cycle analyses (LCA) should be expanded to include the energy costs of advanced processing technologies, ensuring that sustainability benefits are maximized.

AI and machine learning (ML) are transforming the development of HMMA by optimizing texture formation, improving sensory properties, and reducing production costs. ML‐driven techniques facilitate precise ingredient selection, process control, and structural optimization, enabling plant‐based proteins to closely mimic the fibrous texture, juiciness, and mouthfeel of traditional meat products (Kircali Ata et al. 2023). One of the most promising applications of AI in this field is its ability to predict extrusion conditions and ingredient interactions, allowing manufacturers to achieve consistent and meat‐like anisotropic structures. Recent studies have illustrated how AI‐based models can predict and refine textural attributes, improving consumer acceptability and product quality. Another significant advantage of AI integration is flavor and aroma enhancement, particularly for PBMAs. ML models can simulate the Maillard reaction, lipid oxidation, and volatile compound interactions, allowing manufacturers to optimize umami, roasted, and fatty flavor notes (Kircali Ata et al. 2023). Additionally, AI–ML‐based sensory data evaluation can analyze consumer preference data, enabling brands to formulate personalized plant‐based meat alternatives tailored to different market segments (Bi et al. 2020).

Understanding and addressing consumer preferences and perceptions remain critical to the adoption of PBMA. Research into the sensory science of flavor, aroma, and texture perception can guide the design of products that meet consumer expectations. Additionally, exploiting AI to predict and tailor products to consumer preferences based on regional or cultural contexts can further enhance market adaptability. Hybrid models that combine plant‐based proteins with cultured cells or microbial proteins may offer a middle ground for consumers transitioning from traditional meat. These models could combine the sustainability benefits of plant proteins with the sensory and nutritional advantages of cultured cells, creating products that are both innovative and appealing.

## 7 Conclusion

The pursuit of sustainable and health‐conscious alternatives to traditional animal‐based meat has positioned PBMA as a promising solution to the global challenges of food security, environmental sustainability, and public health. This review highlights the critical role of fibrous structure formation in achieving the sensory and textural equivalence of plant‐based products to animal meat. While HME remains the cornerstone technology for creating these fibrous structures, emerging methods such as shear cell technology, 3D printing, and electrospinning offer innovative avenues for further refinement and diversity in product development. Despite significant progress, several challenges persist, including replicating complex anisotropic textures, achieving balanced amino acid profiles, and addressing flavor and juiciness gaps. By emphasizing the customization and cultural adaptability of PBMA, such as incorporating regional flavors and textures, manufacturers can address these barriers and expand their market appeal. Transparent communication about production processes and advancements in taste and texture can enhance consumer trust and adaptability. Furthermore, a deeper understanding of the molecular interactions during processing, coupled with innovative strategies to improve protein functionality, is essential for overcoming these hurdles. The review also underscores the necessity of addressing gaps in consumer acceptance and market adaptation. Factors such as cultural preferences, sensory expectations, and nutritional adequacy must be carefully considered to enhance the appeal of PBMA. Additionally, integrating sustainability metrics into the development process can ensure that these products align with global goals of reducing environmental impact while feeding a growing population.

## Author Contributions

**Saqib Gulzar**: conceptualization, formal analysis, data curation, funding acquisition, writing – original draft, visualization, project administration. **Abdul Fateh Hosseini**: writing – original draft, data curation. **Olga Martín‐Belloso**: writing – review and editing, validation. **Robert Soliva‐Fortuny**: validation, writing – review and editing. **Syed S. H. Rizvi**: supervision; project administration; validation; writing – review and editing.

## Conflicts of Interest

The authors declare no conflicts of interest.

## Data Availability

No data were used for the research described in the article.
